# Planting increases the abundance and structure complexity of soil core functional genes relevant to carbon and nitrogen cycling

**DOI:** 10.1038/srep14345

**Published:** 2015-09-23

**Authors:** Feng Wang, Yuting Liang, Yuji Jiang, Yunfeng Yang, Kai Xue, Jinbo Xiong, Jizhong Zhou, Bo Sun

**Affiliations:** 1 State Key Laboratory of Soil and Sustainable Agriculture, Institute of Soil Science, Chinese Academy of Sciences, No. 71 East Beijing Road, Nanjing 210008, China; 2 Ningbo Academy of Agricultural Sciences, No. 19 Dehou Street, Ningbo 315040, China; 3 State Key Joint Laboratory of Environment Simulation and Pollution Control, School of Environment, Tsinghua University, Beijing 100084, China; 4 Institute for Environmental Genomics and Department of Botany and Microbiology, University of Oklahoma, Norman, OK 73019, USA; 5 Earth Sciences Division, Lawrence Berkeley National Laboratory, Berkeley, CA 94720, USA

## Abstract

Plants have an important impact on soil microbial communities and their functions. However, how plants determine the microbial composition and network interactions is still poorly understood. During a four-year field experiment, we investigated the functional gene composition of three types of soils (Phaeozem, Cambisols and Acrisol) under maize planting and bare fallow regimes located in cold temperate, warm temperate and subtropical regions, respectively. The core genes were identified using high-throughput functional gene microarray (GeoChip 3.0), and functional molecular ecological networks (fMENs) were subsequently developed with the random matrix theory (RMT)-based conceptual framework. Our results demonstrated that planting significantly (*P* < 0.05) increased the gene alpha-diversity in terms of richness and Shannon – Simpson’s indexes for all three types of soils and 83.5% of microbial alpha-diversity can be explained by the plant factor. Moreover, planting had significant impacts on the microbial community structure and the network interactions of the microbial communities. The calculated network complexity was higher under maize planting than under bare fallow regimes. The increase of the functional genes led to an increase in both soil respiration and nitrification potential with maize planting, indicating that changes in the soil microbial communities and network interactions influenced ecological functioning.

Soil microbes comprise a large portion of the genetic diversity and are important components of terrestrial ecosystems, playing a key role in mediating the biogeochemical cycles of carbon (C), nitrogen (N), phosphorus (P), sulfur (S) and various metals^1^. Therefore, it is of great value to profile the soil microbial community structure and its determinants for developing feasible strategies to promote microbial functions in various ecosystems.

It is widely recognized that the aboveground plant strongly impacts the belowground microbial community, which varies with different vegetation types, plant diversity and productivity^2^,^3^. Plants affect the soil microbial community by root exudates, plant litter input and changes in the soil temperature, moisture and biogeochemical properties, especially through rhizodeposition^4^. In agro-ecosystems, long-term cropping changed the soil microbial community structure in comparison with bare fallow^5^. Moreover, the plant genotype altered the structure of rhizospheric microbial communities^6^,^7^. Meanwhile, soil microbial communities are also subjected to the combined influences of many abiotic properties^8^,^9^, fertilization practices^10^,^11^ and climate conditions^12^. Until now, the separation of these influences was difficult and the relative importance of these influences is still unclear. The fallow system is a common farming technique used to raise soil fertility and accumulate soil moisture^13^. The bare fallow (non-planting regime) could be used as a control in the field experiment to separately identify the impact of the crop or fertilization on the soil microbial community structure and functions^5^,^14^.

A core microbiome is typically defined as a suite of members shared among microbial consortia from different habitats, which appears in all assemblages associated with a particular habitat and are likely critical to the function of that type of community^15^. Core microbiomes were studied in forest soil^16^, marsh sediment^17^ and aquatic environments^18^. However, little is known about whether there exists a core determined by plants, and if so, how it contributes to the soil nutrient cycling processes in agricultural soils. Here, we defined ‘core genes’ as those functional genes shared in soils across different geoclimate regions with distinct soil types. This study addressed the questions of whether, and how, plants influence the core genes of soil microbial communities.

The biodiversity of microbial communities includes not only the number of species/populations and their abundance but also the complex interactions (e.g., antagonistic, competitive and mutualistic) among the different species/populations^19^,^20^. Therefore, the network structure formed by the complex species/populations interactions might affect the ecosystem functioning in complex ecosystems^20^,^21^,^22^. Recently, the random matrix theory (RMT)-based approach was proposed and applied to characterize network interactions among different microbial functional groups in response to elevated CO_2_ based on GeoChip hybridization data^22^. The results found that the elevated CO_2_ changed not only the soil microbial community functional structure^23^, but also the functional molecular ecological network (fMEN)^22^. The soil-microbe system is complicated in natural environments^9^; therefore, it is also important to determine whether planting alters the interactions among different microbial functional groups and influences their ecological functions.

There are distinct soil types and climate regimes along a north–south transect of eastern China. We selected three representative zonal soil types along the transect: neutral black soil in the cold temperate zone, alkaline fluvo-aquic soil in the warm temperate zone and acidic red soil in the subtropical zone, covering areas of 7.0 × 10^4^ km^2^, 1.3 × 10^5^ km^2^ and 1.0 × 10^6^ km^2^, respectively. We conducted the investigation in three agricultural experimental stations: the north site in Hailun (N), middle site in Fengqiu (M) and south site in Yingtan (S). The major objectives of the study were: (i) to examine how planting affects the functional microbial community structure in different soil types with different climate regimes, and (ii) to determine if planting has an impact on the functional network structure of soil microbial communities. Our results indicated that planting greatly altered not only the functional gene diversity, but also the complexity of community structure, which might be useful for predicting microbial ecological functions responses to planting.

## Results

### Soil geochemical properties

**Figure 1:**
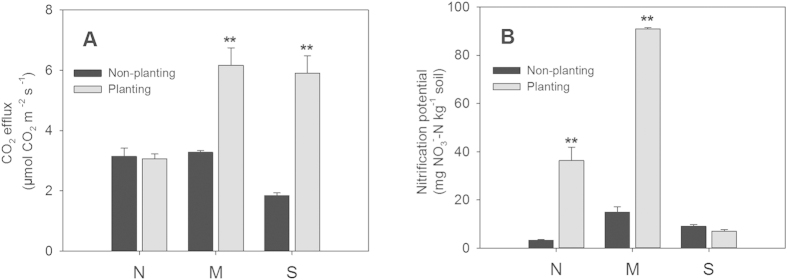
Mean CO_2_ efflux (A) and nitrification potential (B) in three soils under the non-planting and planting treatments. N, north site in Hailun with cold temperate climate; M, middle site in Fengqiu with warm temperate climate; and S, south site in Yingtan with subtropical climate. All data are presented as the mean ± SE. **denotes a significant difference at *P* < 0.01 assessed using a two-tailed t test.

There was a large variation in the soil geochemical properties at the three sites. The contents of total phosphorus (TP), total potassium (TK), available nitrogen (AN), available phosphorus (AP), available potassium (AK), NH_3_-N, NO_3_-N and soil pH were altered to some extent by planting, although planting effects differed at the three locations. For example, the soil pH and TK content were significantly (*P* < 0.05) altered by planting at M and S, but not in N (Table 1). Planting significantly increased the CO_2_ efflux (*P* < 0.01) by 88% and 222% at M and S (Fig. 1A), respectively. Planting increased the soil nitrification potential by 5- and 10-fold at M and N (Fig. 1B), respectively. The The CO_2_ efflux showed significant correlations with the aboveground biomass (r^2^ = 0.597, *P* < 0.01) and the chlorophyll concentration (r^2^ = 0.737, *P* < 0.01) but not with the soil chemical properties.

### Functional gene structure

A total of 8870 genes after data normalization and processing belonging to 259 gene families were detected by GeoChip 3.0. The calculated functional genes α-diversity indices, including richness, Shannon’s (*H*′) and Simpson’s (*1/D*), were found to be significantly more diverse (*P* < 0.05) in planting than non-planting conditions across all of the tested soil types (Figure S1, Figure S2). However, the soil microbial biomass, as measured by phospholipid fatty acids analysis (PLFA), was not changed by planting in N and S, but significantly increased in M (Fig. S3).

To further assess the relative effects of planting and location factors on functional gene α-diversity, the Geochip data presented above were subjected to a multivariate regression tree (MRT) analysis. The MRT tree was first split by planting and then by location (Fig. S4), which explained 83.5% and 12.8% of the variation of the soil functional gene diversity, respectively. In accordance, a non-parametric multivariate analysis of variance test (adonis) indicated that the microbial community functional structures significantly (*P* < 0.05) differed between the planting and non-planting treatments (Table S1).

**Figure 2:**
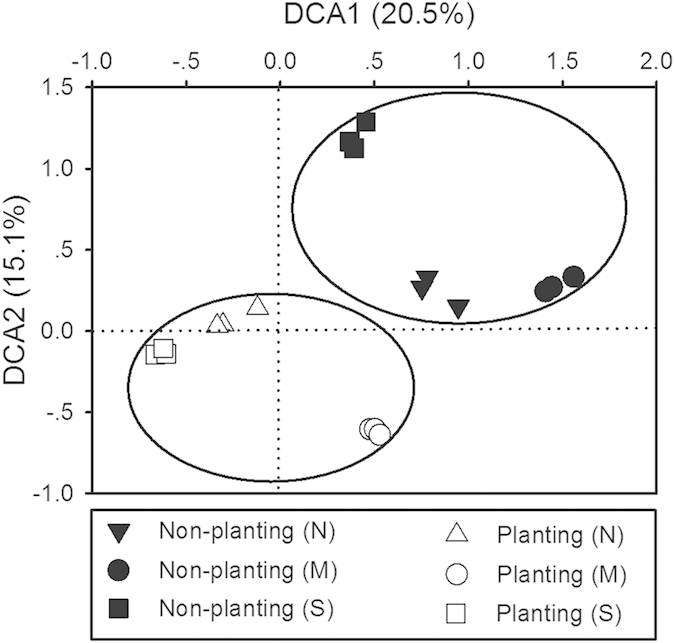
Detrended correspondence analysis (DCA) for all of the detected functional genes from three soils under the non-planting and planting treatments. N, north site in Hailun with cold temperate climate; M, middle site in Fengqiu with warm temperate climate; and S, south site in Yingtan with subtropical climate.

Moreover, the detrended correspondence analysis (DCA) results clearly showed that the soil microbial functional genes were grouped primarily based on planting rather than their locations (Fig. 2). Interestingly, there was a larger difference in the microbial functional genes between the planted and unplanted soils in the M and S sites than in the N site. Among the three sites, the soil microbial functional genes were more similar between the N and M sites for the non-planting treatment, and between N and S for the planting treatment. The DCA provided strong evidence that the structures of the microbial functional genes were determined by planting and locations, with planting as the primary driving factor.

### Core functional genes

**Figure 3:**
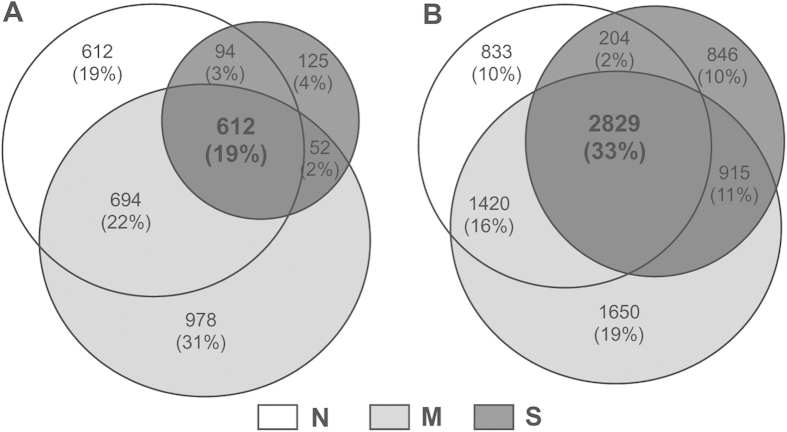
Venn diagrams based on all of the detected functional genes used to visualize gene distribution and overlap among three soils under the non-planting (A) and planting treatments (B). The numbers of detected genes in each part with their percentages in brackets are indicated. The relationships within and among soils are scaled relative to the total gene numbers. N, north site in Hailun with cold temperate climate; M, middle site in Fengqiu with warm temperate climate; and S, south site in Yingtan with subtropical climate.

The core genes were denoted as the microbial functional genes present in all three of the soils. Only 612 core genes (19% of the total) were identified in the unplanted soils; while 2829 (33% of the total) were found in all of the maize-planted soils (Fig. 3), including 597 core genes of the unplanted soils. Therefore, the emergence of a total of 2232 core genes represented those influenced by planting across all of the measured soil types. These genes belonged to a variety of functional categories of organic remediation, antibiotics, metal resistance, and C, N, P and S cycling. Because C degradation and N-cycling microbial communities have attracted more attention in the field of agriculture and environmental protection, the following study is only concerned with those genes.

A total of 272 C degradation genes were among the core genes attributed to the planting effects (Table S2). These genes were distributed in the degradation of relatively labile C (e.g., starch, hemicelluloses, cellulose and simple aromatics) and recalcitrant C (e.g., lignin), suggesting that planting affected all of the C degradation processes. Notably, *aceA* and *aceB* were two genes involved in the utilization of acetate. Their abundances accounted for a higher proportion (21.9%) of C cycling genes in the core than in the total detected gene pools (17.5%), indicating that C input from plants (especially intermediates of plant metabolism) stimulated microorganisms functional potentials.

Forty percent of the core C degradation genes were related to Proteobacteria. Many of these genes were related to rhizosphere microorganisms, such as *amyA* of starch degradation related to *Rhodopseudomonaspalustris* and *Enterobacter* sp., *bgl* of cellulose degradation from *Agrobacterium* sp. and *chi* (endochitinase) of chitin degradation from *Bacillus circulans*. In addition, *pulA* encoding pullulanase from *Sinorhizobium meliloti* 1021 was detected in all of the planted soils but not in the unplanted soils. *Sinorhizobium meliloti* is an α-Proteobacterium that alternates between a free-living phase in bulk soil or rhizosphere and a symbiotic phase within host plant cells^24^.

The GeoChip 3.0 contains 16 gene families to characterize the different N cycling processes. A total of 241 genes distributed over 15 families (except *hao*) of N cycling genes were found in the core (Table S3). The genes enriched in the core, when compared with the total detected genes of all samples, included *narG* involved in denitrification, *ureC* involved in ammonification and *nifH* involved in N_2_ fixation. In contrast, the percentages of *amoA* involved in nitrification and *nirK*/*nirS* involved in denitrification were lower in the core than the total detected genes.

A number of core N cycling genes were related to the plant pathogens and plant symbiotic microorganisms, e.g., *nifH* genes from both the free-living and symbiotic N-fixing microorganisms, including Proteobacteria, Firmicutes, Archaea, Chlorobi and Cyanobacteria. There were *ureC* genes related to Rhizobium (i.e., *Sinorhizobium medicae* and *Rhodopseudomonas palustris*) and *Pseudomonas* (i.e., *Pseudomonas fluorescens*), which are considered to be intimately associated with plants and benefit to plant growth^25^.

### Relationships between core genes and environmental variables

The partial Mantel test indicated that all of the functional genes detected in unplanted soils were significantly correlated (*P* < 0.05) with the soil and climate variables, such as soil pH, TK, NH_4_-N, annual rainfall (AR) and relative humidity (RH) (Table 2). In addition to the soil and climate factors, the genes detected in maize-planted soils were also significantly correlated (*P* < 0.01) with plant variables, such as the aboveground biomass (AGB) and the plant leaves chlorophyll concentration (SPAD). The core genes in the unplanted soils showed no significant correlation with any environmental variables, while the increased core genes were significantly (*P* < 0.05) correlated with three plant-growth variables (AGB, SPAD and soil moisture). Together, these results suggested that the increased core genes were influenced by planting but were independent of the location representing soil type and climate regime.

Correlation analyses were used to examine the influence of the aboveground biomass on individual functional genes. The aboveground biomass was significantly (*P* < 0.01) correlated with ten genes involved in C degradation (Table S4). Only three genes involved in the degradation of relatively labile C (e.g., starch and hemicelluloses), including those encoding alpha amylase (*amyA*), pullulanase (*pula*) and xylanase (*xyn*), were correlated with the aboveground biomass. However, five genes involved in the recalcitrant carbon degradation (chitin and lignin), including those encoding endochitinase (*chi*) and phenol oxidase (*phox*), were correlated with the aboveground biomass. Notably, three out of four *chi* genes were related to Ascomycota. For the nitrogen cycling, only eight genes were correlated with the aboveground biomass (Table S4), and they were associated with nitrogen fixation (*nifH*), dissimilatory N reduction (*napA* and *nrfA*) and ammonification (*ureC*). In contrast, no denitrification genes (e.g., *nirK* and *nirS*) were correlated with the aboveground biomass, despite the presence of a large number of those probes on GeoChip. Interestingly, all of the C degradation and N cycling genes were positively correlated with the aboveground biomass, suggesting that the maize biomass increased gene abundance and microbial functional potentials.

### Specific core genes linked to soil respiration and nitrification potential

A total of ten C degradation genes and ten N cycling genes were significantly (*P* < 0.05) correlated with soil respiration and nitrification potential (Table 3), respectively. The C degradation genes were distributed in both the labile and recalcitrant C degradation, suggesting that soil respiration was determined by both labile and recalcitrant C degradation microbes. Additionally, one chitin degradation gene (*chi*) related to *Hypocreavirens* correlated with both soil respiration and the aboveground biomass. Only one individual *amoA* gene from an uncultured bacteria was significantly (*P* < 0.05) correlated with nitrification potential. The results were consistent with a previous report of ammonia-oxidizing bacteria playing a crucial role in controlling nitrification in agricultural soil^26^. In addition to the *amoA* gene, there were genes involved in N fixation, denitrification, ammonification and dissimilatory N reduction, indicating an interlinked N cycling process in the soils.

### Network structure

To further understand whether planting affected the ecological interactions among the different microbial functional groups, six networks of different microbial communities were constructed by the RMT-based approach with whole genes, C cycling and N cycling genes under planting and non-planting treatments (Table 4). The number of nodes (network size) and total links of fMENs were considerably higher under the planting treatment than the non-planting treatment (Table 4). Notably, two whole fMENs only shared 22.1% (241) of their nodes, and the network structure composition of the key functional genes was substantially different between the two treatments (Figure S5). The fMENs under maize planting had higher connectivity (avgK) and modularity in three pairs of networks at the individual gene level when compared to the non-planting treatment.

**Figure 4:**
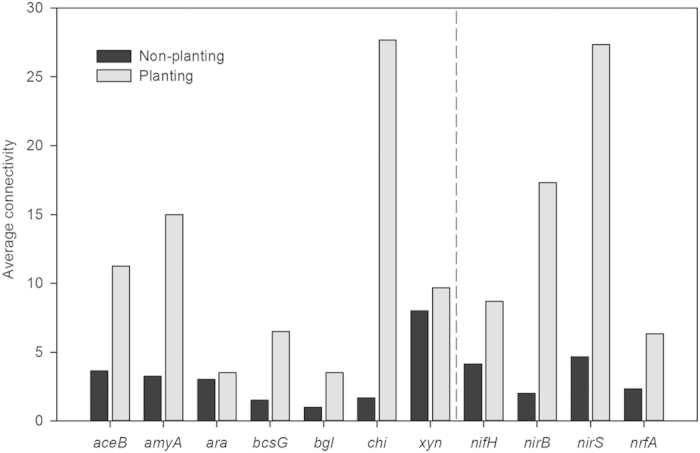
The average connectivity of the major functional genes shared in two whole fMENs under the non-planting and planting treatments. The listed functional genes for C and N cycling include *aceB* (malate synthase), *amyA* (alpha amylase), *ara* (arabinofuranosidase), *bcsG* (endoglucanase), *bgl* (cellobiase), *chi* (endochitinase), *xyn* (xylanase), *nifH* (nitrogenase reductase), *nirB* (nitrite reductase), *nirS* (nitrite reductase) and *nrfA* (c-type cytochrome nitrite reductase).

The network complexities measured by the average connectivity of the shared nodes were also compared at the functional gene group level. For the whole fMENs, the network complexity for most of the functional gene groups was generally higher under the planting treatment (Fig. 4). For example, the average connectivity of nodes for carbon degradation (*aceB*, *amyA*, *bcsG*, *bgl*, and *chi*) and denitrification (*nirS*) were substantially higher under the planting treatment. These results suggested that planting substantially altered the network structure among the various microbial functional gene groups.

## Discussion

Although the plant species richness could regulate the belowground microbial diversity and community composition, it was reported that soil microbial diversity might not be increased by planting crops^5^,^14^,^27^ when using denaturing gradient gel electrophoresis (DGGE), PLFA and Biolog analysis. Similarly, the *narG* gene diversity was not significantly different between the unplanted and maize-planted soils analyzed by restriction fragment length polymorphism^28^. In the present study, planting significantly increased the functional gene α-diversity across all of the soil types, according to the high-throughput comprehensive microarray method. Subsequent DGGE analysis also confirmed that planting significantly increased band number by 15%–26% (Figure S1). Additionally, the Shannon and Simpson indices showed more diversity in the planting treatments than the non-planting treatments (Figure S2). Therefore, we concluded that planting consistently increased the functional and taxonomical diversity across distinct soil types.

There were three assumptions to explain why planting increased the diversity of soil microbial communities. First, planting increased soil physical heterogeneity by changing the soil physical structures such as soil temperature, moisture, porosity, particles and diffusional characteristics, and resulted in more soil microhabitat for microorganisms^9^. Second, plant rhizodeposits supplied nutrients (e.g., sugars, polysaccharides, amino acids, peptides, proteins and fatty acids) to support larger numbers and specialized soil microorganisms^2^,^3^. For instance, α-*Proteobacteria*^29^ and the fungal phyla Ascomycota and Glomeromycota can respond rapidly to rhizodeposits^30^,^31^. Finally, plant-secreted chemical signaling compounds mediate the interactions of plants with their associated microbiota. For example, strigolactones stimulate the growth of arbuscular mycorrhizal fungi^32^ and benzoxazinoids in maize root exudates attract *Pseudomonas*^33^. It should be noted that a small amount of microorganisms may be brought into soils through the maize seeds. However, recent work found that bacterial numbers and diversity were much higher at the rhizosphere than at the maize root inner tissues^34^, which, to some extent, proved the validity of our findings.

The two-way analysis of variance (ANOVA) showed that the total number of detected functional genes (F value = 44.57) were significantly (*P* < 0.01) different in the three sites. In addition, some unique genes were only harbored in one type of soil, with an average of 18% and 13% of them in unplanted and planted soils, respectively (Fig. 3). These results confirmed the previous reports that historical contingencies of soil types and climate regimes affected microbial communities^35^,^36^. This was further verified in our study by partial Mantel tests that revealed positive correlations with the microbial community structure for soil (pH, TK and NH_4_-N) and climate factors (AR and RH) (Table 2).

There is an increasing awareness that the soil microbial community composition diverges among distant sites, as observed in macroorganisms^37^,^38^. The percentage of core genes in all of the detected genes increased from 19% in the unplanted soils to 33% in the planted soils (Fig. 3), indicating the contemporary disturbance of planting enlarged the core functional microbial community. Plants can increase the soil physico-chemical heterogeneity and result in a number of new niches which support more types of soil microorganisms^2^,^3^,^9^. The increased grass biomass under pulsed nutrient enrichment could raise the species number of arthropod herbivores and carnivores^39^. Another possibility was that a very large pool of dormant microorganisms with a broad spectrum of potential metabolic activities provided a quick growth response to the input of signaling molecules and easily decomposable substrates from plant rhizodeposits^4^. The core genes associated with planting were involved in various biogeochemical processes and belonged to many phylogenetic groups. This knowledge gives a deeper understanding of the common influence on microbial communities and their potential impacts on ecosystem functioning across different soil types^15^.

Traditionally, most microbial diversity studies only just pay attention to species and the abundance of each species, but not their interactions^21^,^22^. When compared to species diversity, network interactions could be more important to ecosystem processes and functions, especially soil microbial communities in natural ecosystems given their extreme complexity, high diversity, and as-yet-uncultivated status^40^. Metagenomic technologies, such as high-throughput microarrays, can offer sufficient and comprehensive experimental data for characterizing network interactions among different functional groups /populations^41^,^42^. In this study, the average connectivity and modularity of these fMENs were significantly different between the non-planting and planting treatments (Table 4). A module denotes a group of species that interact strongly among themselves but interact little with species in other modules. More modules in fMENs under planting may reflect a higher degree of habitat heterogeneity in maize planted soils. It should be noted that the network interactions of the functional genes may cause more complex relationships between the microbial community structure and soil processes^22^.

The soil nitrification potentials increased with planting in the M and N sites, but they were unchanged in the S site (Fig. 1B). The increased soil functional and taxonomical diversities with planting generally increased the soil microbial activities. However, the composition of the preferential species of nitrifying bacteria changed with planting and consequently affected the nitrification potential. A 16-year field experiment showed that all of the ammonia-oxidizing bacteria (AOB) sequences in upland red soils were affiliated with *Nitrosospira* or *Nitrosospira*-like species. Their distribution changed from 16% of cluster 3a and 3b and 84% of cluster 3c under bare fallow to 100% of cluster 3c under wheat–maize rotation^43^. On the contrary, the mean soil pH decreased from 5.97 under bare fallow to 5.09 under the maize planting treatment in the south site. The lower soil pH could decrease the AOB abundance and prevent soil nitrification activity from increasing with the genetic diversity^44^.

In comparison with the phylogenetic gene marker (i.e., 16S-based studies), the GeoChip is advantageous in targeting a wide array of key genes involved in microbial functional processes of interest, such as the biogeochemical cycling of carbon and nitrogen^41^,^42^. Our results showed that the increased gene richness (Figure S1A) coincided with higher soil respiration under planting in the middle and south sites, but not in the north site (Fig. 1A). The relationships between biodiversity and ecosystem functioning could be explained by the species redundancy or compensatory hypothesis^45^,^46^, which means that different species perform the same functional role in ecosystems so that changes in species diversity does not affect the ecosystem functioning. The ecosystem functioning (e.g., biomass or nutrient retention) could be maximized at the lower levels of species richness^47^. As the species interactions were shaped by the abiotic environment, the higher nutrient contents in the black soil in the north site, when compared with the middle and south sites, resulted in a high soil microbial species richness, which in turn resulted in an equivalent soil respiration between the non-planted and planted soils. Consequently, the disturbed community was being functionally similar to the original community and led to unchanged soil respiration. The functional genes detected by GeoChip 3.0 only indicated the potential presence of the corresponding function, which were several steps away from the actual metabolic activity^28^. Therefore, it was difficult to ascribe a microbial process to an active population of microorganisms using a single technique. In addition, the low abundance of *amoA* may indicate that *hao* encoding hydroxylamine oxidoreductase was below the limit of detection of GeoChip. Further integration of the GeoChip with other methods, such as transcriptomics and stable isotope probing approaches is needed.

In conclusion, by combining the high-throughput microarray hybridization technique and novel RMT-based network approach, this study examined the effects of maize planting on microbial community functional structure and network interactions in three zonal soils at a large spatial scale. The functional structure of the microbial community and the network interactions were distinctly different between the non-planting and planting soil treatments. Maize planting increased soil functional gene diversity and the fMENs complexity. To our knowledge, this is the first study to demonstrate the changes in both soil microbial community functional structure and network structure in response to planting. Thus, it could provide a better understanding of the functional traits of the microbial community to help predict ecosystem processes under changing environmental conditions.

## Methods

### Experimental site and sampling

In 2005, a closely controlled field experiment was set up at Hailun (N), Fengqiu (M) and Yingtan (S) Agricultural Experiment Stations in the Chinese Ecosystem Research Network across the north–south transect of eastern China. There are substantial differences in the climate regimes and soil types among these three sites. The mean annual temperature and precipitation at the three sites increases from north to south (Table S5). The soil types at N, M and S sites are neutral black soil derived from loamy loess, alkaline fluvo-aquic soil derived from alluvial sediments of the Yellow River, and acidic red soil derived from Quaternary red clay, which belong to Udic Isogumosol, Aquic Cambisol, Udic Ferralsol in Chinese Soil Taxonomic Classification^48^, and Phaeozem, Cambisol and Acrisol in FAO soil classification system^49^, respectively.

At each experiment site, six plots of 1.2 × 1.4 m^2^ were established in an upland field, and each plot was separated from the adjacent plot using a cement mortar brick wall to a depth of 1 m. Two treatments with 3 replications were applied, including planting and non-planting (bare fallow) treatments. The soils in this experiment all were planted with maize for long term (>20 years) prior to setting up the experiments. In the planting treatment, the common maize cultivars were planted annually in each plot with a 20 cm × 30 cm interval, which were *Zea mays* L. cv. Haiyu 6, Zhengdan 958 and Chenghai 11 in N, M and S sites, respectively. Commercial seeds were coated with mixed materials containing trace amount of insecticides, fungicides, etc. to improve the seed germination rate. Conventional tillage practices and direct seeding were carried out in this experiment. The aboveground biomass of maize was removed from plots at maturity, but the roots were left in the soils. During the whole experiment, no fertilizer and agrochemicals (insecticides, fungicides) were applied, and regular de-weeding (hand pulling) was conducted in both treatments.

Soil samples were taken from the top 15 cm of each plot with a stainless steel cylinder (3 cm inner diameter) one week after the harvest of maize in 2009. Ten soil cores was collected in an S-shaped transect from each plot and then mixed to form one composite sample with weight of approximatedly 300 g. The samples were immediately shipped to the laboratory on ice packs, and then sieved through a 2 mm mesh to remove roots and other debris. A subsample of the sieved soils was stored at −80 °C for genomic DNA extraction. The other parts of the sieved soil samples were stored at 4 °C for nitrification potential and mineral N content analyses. The rest of the soil samples were air dried to determine the soil physicochemical properties.

### Soil geochemical properties, meteorological data and vegetation

Soil pH was determined with a glass electrode (Mettler Toledo Instruments, Shanghai, China) using a soil-to-water ratio of 1:2.5. The soil organic matter (SOM) and the total nitrogen (TN) were determined by dichromate oxidation and Kjeldahl digestion, respectively. The available nitrogen (AN) was measured using the Illinois Soil Nitrogen Test diffusion method. The soil mineral nitrogen was extracted with 2 mol L^−1^ KCl in a 1:4 soil-to-solution ratio for 1 h. NH_4_-N and NO_3_-N in the extracts were determined by Auto Analyzer 3 (Bran+Luebbe GmbH, Germany). The soil total phosphorus (TP) content was analyzed after fusion with sodium carbonate, and the soil available phosphorus (AP) was extracted by sodium bicarbonate and determined using the molybdenum blue method. The soil total potassium (TK) was extracted by fusion with sodium hydroxide, and the soil available potassium (AK) was extracted by ammonium acetate and determined by flame photometry (CANY Precision Instrument Co., Ltd, Shanghai, China).

The aboveground biomass was determined by the oven drying method (80 °C drying to constant weight). The SPAD (plant leaves chlorophyll concentration) was collected during the maize tasseling stage in 2009, with the average of five leaves’ data. The mean annual temperature, precipitation and relative humidity data were collected from the three experiment stations in 2009.

### Soil nitrification potential and CO_2_ efflux

The soil nitrification potential was measured by the previous method with some modifications^50^. From each sample, 10 g (oven-dried weight equivalent) soil was added into a 100 ml bottle. Deionized water was added to adjust the soil moisture content to 60% of the water holding capacity. The 10 ml (NH_4_)_2_SO_4_ solution was applied at rates of 0 (i.e., control) or 200 mg N kg^−1^. All of the bottles were covered with aluminum foil punctured with needle holes to maintain an aerobic condition, but prevent water evaporation. The bottles were incubated at 28 °C. The loss of water was replenished using deionized water every 1 d. Samples were collected at 0 and 14 days after the beginning of the incubation. NO_3_-N in the soil was extracted with 10% KCl for 1 h and determined by the continuous flow autoanalyzer (Bran+Luebbe GmbH, Germany). The changes of NO_3_-N were defined as the soil nitrification potential.

The soil CO_2_ efflux and soil temperature were measured weekly during the maize growing season using LI-6400 Portable Photosynthesis System (LI-COR Inc., Lincoln, Nebraska, USA). PVC collars (10 cm long, 10 cm inside diameter) were inserted into the inter-row soils at 5 cm depth at least 24 h prior to measurement. The soil respiration chamber was set on top of these collars following the protocol recommended by the LI-6400 manual to measure undisturbed soil CO_2_ efflux.

### Phospholipid fatty acids analysis

The soil microbial biomass was estimated with 2.0 g of soil by phospholipid fatty acids analysis (PLFA), using a modified Bligh-Dyer technique as described previously^51^. The extracted PLFAs were analyzed by the MIDI Sherlock Microbial Identification System (MIDI, Newark, DE, USA). The 19:0 fatty acid methyl ester (FAME) was added to each sample as an internal standard to quantify each PLFA contents based on their peak area.

### GeoChip 3.0 analysis

DNA was extracted from 5 g of soil and purified with the freeze-grinding mechanical lysis method^52^. Next, 2 μg of purified DNA from each soil sample was used for GeoChip 3.0 hybridization in triplicates. The DNA (2 μg) was labeled with the fluorescent dye Cy-5 using random priming. After labeling, the labeled target was resuspended in a hybridization solution, denatured at 95 °C for 5 min and deposited at 50 °C. In addition, a 50-mer common oligonucleotide reference standard as mixed with all these probes, including geneprobes and controls, and co-spotted on GeoChip 3.0 as a common reference standard for data normalization and comparison^53^. Hybridization was performed in triplicates with a TECAN Hybridization Station HS4800 Pro (TECAN, US) according to the manufacturer’s protocol. Then a Scan Array Express Microarray Scanner (Perkin Elmer, Boston, MA) was used to scan the microarray, and the ImaGene version 6.0 (Biodiscovery, El Segundo, CA) was used to determine the intensity of each spot and identify poor-quality spots.

GeoChip analyses were carried out by the Microarray Data Manager on the website (http://ieg.ou.edu/microarray/). The analysis pipeline^41^ contained the following major steps. First, we removed the poor-quality spots. The spots flagged as 1 or 3 and with a signal to noise ratio (SNR) less than 2.0 were removed. Second, we normalized the raw data at three levels: individual sub-grids on a single slide, technical replicates among samples and across the whole data set. Third, we removed the outliers. Replicates (signal–mean) were removed when they had more than two times the standard deviation. This process continued until no such replicates were identified. Fourth, at least 0.34 time of the final positive spots (probes), or a minimum of two spots, were required for each gene to be considered for data analysis. Finally, we removal the probes only detected in one sample among the three biological replicates for data reliability. Subsequently, the relative abundance was calculated in each sample and then multiplied by the mean value for the sums of signal intensity in all of the samples. A natural logarithm transformation was performed for the amplified relative abundance plus 1.

### Denaturing gradient gel electrophoresis

General primers PRBA338F with GC clamp and PRUN518R were used to amplify the variable V3 region of the bacterial 16S rRNA genes^54^. The resultant PCR products were subject to polyacrylamide gel electrophoresis with a denaturing gradient of 35–60%. Ribosomal genotypes and their relative abundance were estimated using band position and intensity, which were analyzed with the help of the Quantity One program (Bio-Rad Laboratories, Hercules, CA).

### Statistics

The Shannon index (*H*′) (Equation 1) and Simpson index (1/D) (Equation 2) were calculated using the following equations:


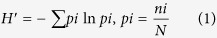



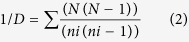


Where *pi* is the proportional signal intensity of the *i*th gene relative to the total signal intensity (*N*) of all the detected genes.

The Richness, Shannon and Simpson indices were selected to reflect the microbial diversity, and they were analyzed by Krebs/win version 0.94 (http://www.biology.ualberta.ca/jbrzusto/krebswin.html).

A multivariate regression tree (MRT) was conducted to partition the relative importance of planting and location on the functional genes diversity. The impurity of a node was defined as the total sum of squares about the multivariate mean, and each split minimized the total sums of squared distances (SSD, Euclidean distances) of sites from the centroids within two nodes, and maximized the SSD between the node centroids^55^. A non-parametric multivariate analysis of variance (adonis) using the Bray-Curtis similarity index was used to calculate a distance matrix from GeoChip data based on the dissimilarities among samples. The Monte Carlo permutation was used to test the significance of the statistics. A detrended correspondence analysis (DCA) was conducted to explore the overall functional changes in the microbial communities. A partial Mantel test was conducted to evaluate the possible linkages between the community structure and environmental variables. The Bray-Curtis coefficient and Euclidean distance were used to construct dissimilarity matrices of the communities and environmental variables, respectively. All of the above analyses were performed in the R statistical programming version 2.13.2. The MRT was conducted using the package of ‘mvpart’ (http://cran.r-project.org/web/packages/mvpart/). The adonis, DCA and partial Mantel test were conducted using the package of ‘Vegan’ (http://vegan.r-forge.r-project.org/).

A one-way ANOVA was used to determine the differences between the diagnostic groups. Student’s t-tests were used to test the significance of the differences between the planting and non-planting. A correlation analysis using Pearson’s method was used to test the significance correlation between two variables. All of these methods were performed using the SPSS version 16.0 (SPSS Inc., Chicago, Illinois, USA).

### Network construction and characterization

Six functional molecular ecological networks (fMENs) were constructed by the RMT-based approach with the log transformed hybridization data, as previously described^21^,^22^,^56^. First, the Pearson correlation analysis between individual genes was calculated. The correlation matrix was subsequently converted to a similarity matrix, which measured the degree of concordance between the abundance profiles of the genes across different samples. An adjacency matrix was generated from the similarity matrix by using an appropriate threshold (S_t_) for defining the network structure. In our study, N cycling fMENs under planting and non-planting conditions were constructed with an identical similarity threshold (S_t_) 0.86 (Table 4). For the whole fMENs and C cycling fMENs, very close similarity threshold were obtained for non-planting and planting. The S_t_ values in these networks were higher than most of the previous networks based on the RMT approach^22^,^56^, suggesting that the six constructed networks in the present study were applicable to the following network analysis.

Cytoscape 2.6.3 software was used to visualize the network structure graphs and other gene information such as taxonomy, edge information. Two indexes were used to describe the overall topology structure of the different networks: (i) average connectivity (*avgK*), which was used to describe network complexity, higher *avgK* means a more complex network; (ii) modularity, which was used to describe how well the modules are separated, each network was separated into modules which were usually considered as functional units in biological systems^57^. Network construction and network properties calculation^56^ were carried out on molecular ecological network analysis pipeline (http://ieg2.ou.edu/MENA/).

## Additional Information

**How to cite this article**: Wang, F. *et al.* Planting increases the abundance and structure complexity of soil core functional genes relevant to carbon and nitrogen cycling. *Sci. Rep.* **5**, 14345; doi: 10.1038/srep14345 (2015).

## Supplementary Material

Supplementary Information

## Figures and Tables

**Table 1:** Soil geochemical properties in three sites under non-planting and planting treatments^a^.

Soil property^b^	Non-planting (N)^c^	Planting (N)	Non-planting (M)	Planting (M)	Non-planting (S)	Planting (S)
pH	6.26 ± 0.10c	6.28 ± 0.02c	7.70 ± 0.12b	8.68 ± 0.03a	5.97 ± 0.22c	5.09 ± 0.06d
SOM (g kg^−1^)	48.57 ± 2.94a	48.85 ± 0.81a	9.49 ± 0.32b	7.39 ± 0.40b	9.92 ± 0.41b	10.50 ± 0.40b
TN (g kg^−1^)	2.13 ± 0.15a	2.28 ± 0.08a	0.61 ± 0.06b	0.59 ± 0.06b	0.64 ± 0.02b	0.74 ± 0.06b
TP (g kg^−1^)	0.83 ± 0.02b	0.91 ± 0.02a	0.62 ± 0.02c	0.57 ± 0.02c	0.44 ± 0.00d	0.45 ± 0.02d
TK (g kg^−1^)	18.53 ± 0.19a	18.05 ± 0.13a	17.84 ± 0.17a	16.64 ± 0.42b	9.86 ± 0.12c	8.86 ± 0.22d
AN (mg kg^−1^)	161.7 ± 3.8b	206.8 ± 4.8a	41.8 ± 1.1d	35.2 ± 1.1d	52.8 ± 0.0c	57.2 ± 1.1c
AP (mg kg^−1^)	33.04 ± 5.82a	25.57 ± 2.12ab	6.58 ± 0.26c	2.26 ± 0.47c	21.06 ± 1.32b	20.32 ± 1.33b
AK (mg kg^−1^)	152.5 ± 1.4a	150.8 ± 6.0a	95.8 ± 5.1c	62.5 ± 5.2d	131.3 ± 3.6b	143.3 ± 5.8ab
NO_3_-N (mg kg^−1^)	16.69 ± 0.31a	6.98 ± 0.32b	3.50 ± 0.16d	4.15 ± 0.32d	5.29 ± 0.17c	5.24 ± 0.21c
NH_4_-N (mg kg^−1^)	0.32 ± 0.04d	0.62 ± 0.05c	0.79 ± 0.01b	0.78 ± 0.08b	1.51 ± 0.01a	1.62 ± 0.04a

^a^All data are presented with mean ± SE, and values within the same row followed by the different letter indicate a significant difference at *P* < 0.05.

^b^Abbreviations: SOM, soil organic matter; TN, total nitrogen; TP, total phosphorus; TK, total potassium; AN, available nitrogen; AP, available phosphorus; AK, available potassium.

^c^N, north site with cold temperate climate; M, middle site with warm temperate climate; S, South site with subtropical climate.

**Table 2:** Correlations between environmental variables and soil microbial community functional structures assessed by partial Mantel tests.

Variables^a^	Non-planting^b^		Planting^c^		Non-planting core^d^		Increased core by planting^e^	
	r	*P*	r	*P*	r	*P*	r	*P*
pH	0.442	**0.015**	0.673	**0.000**	0.237	0.135	0.025	0.305
SOM	−0.577	0.999	−0.380	0.999	−0.168	0.763	−0.287	0.999
TN	−0.619	1.000	−0.457	1.000	−0.098	0.601	−0.259	0.999
TP	−0.135	0.811	−0.295	0.983	−0.214	0.884	−0.292	1.000
TK	0.547	**0.013**	0.431	**0.018**	−0.046	0.443	−0.062	0.847
AN	−0.647	1.000	−0.393	1.000	−0.190	0.844	−0.235	0.998
AP	0.027	0.402	0.241	**0.037**	0.282	0.167	0.013	0.373
AK	−0.014	0.455	0.386	**0.026**	0.333	0.103	−0.047	0.763
NO_3_-N	−0.649	0.999	−0.076	0.713	−0.155	0.811	0.051	0.173
NH_4_-N	0.289	**0.026**	0.435	**0.026**	−0.160	0.753	−0.115	0.956
MSM	0.632	**0.001**	0.225	0.062	0.212	0.151	0.199	**0.034**
MST	−0.690	1.000	−0.254	0.948	−0.207	0.891	0.037	0.231
AGB	—	—	0.451	**0.000**	—	—	0.145	**0.044**
SPAD	—	—	0.345	**0.000**	—	—	0.764	**0.000**
MAT	−0.569	0.998	−0.234	0.955	−0.298	0.946	−0.363	1.000
AR	0.597	**0.009**	0.525	**0.012**	−0.018	0.441	−0.073	0.887
RH	0.527	**0.014**	0.459	**0.018**	−0.063	0.511	−0.128	0.983

^a^Abbreviations: SOM, soil organic matter; TN, total nitrogen; TP, total phosphorus; TK, total potassium; AN, available nitrogen; AP, available phosphorus; AK, available potassium; MSM, mean soil moisture; MST, mean soil temperature; SPAD, chlorophyll concentration of plant leaf; AGB, aboveground biomass; MAT, mean annual temperature AR , annual rainfall; RH, relative humidity.

^b^Total detected functional genes in three soils under non-planting treatment.

^c^Total detected functional genes in three soils under planting treatment.

^d^Shared functional genes among three soils under non-planting treatment.

^e^Shared functional genes among three soils under planting treatment in which the non-planting core was removed.

**Table 3:** Core functional genes involved in carbon degradation or nitrogen cycling correlated significantly (*P* < 0.05) with CO_2_ efflux or soil nitrification potential^a^.

Genbank. ID	Gene^b^	Sub-category	Organism
92441508	*amyA*	Starch degradataion	Mycobacterium sp. KMS
49613597	*amyA*	Starch degradataion	Erwinia carotovora subsp. atroseptica SCRI1043
154304204	*ara*	Hemicellulose degradataion	Botryotinia fuckeliana B05.10
145596104	*xylA*	Hemicellulose degradataion	Salinispora tropica CNB-440
114050057	*vanA*	Aromatics degradataion	Streptomyces ambofaciens
218758227	*aceB*	Aromatics degradataion	Desulfovibrio vulgaris str. ‘Miyazaki F'
**113129062**	***chi***	**Chitin degradataion**	**Hypocrea virens**
119189427	*chi*	Chitin degradataion	Coccidioides immitis RS
83815976	acetylglucosaminidase	Chitin degradataion	Salinibacter ruber DSM 13855
598355	*lip*	Lignin degradataion	Phanerochaete chrysosporium
71361353	*amoA*	Nitrification	uncultured bacterium
125714545	*ureC*	Ammonification	Clostridium thermocellum ATCC 27405
112462455	*nirK*	Denitrification	uncultured bacterium
12744271	*nosZ*	Denitrification	uncultured temperate forest soil bacterium CZ1441
**124488109**	***napA***	**Dissimilatory N reduction**	**uncultured bacterium**
**95133480**	***nrfA***	**Dissimilatory N reduction**	**Desulfuromonas acetoxidans DSM 684**
154151622	*nifH*	Nitrogen fixation	Candidatus Methanoregula boonei 6A8
**3157614**	***nifH***	**Nitrogen fixation**	**unidentified nitrogen-fixing bacteria**
**148342406**	***nifH***	**Nitrogen fixation**	**uncultured bacterium**
**2897667**	***nifH***	**Nitrogen fixation**	**Paenibacillus macerans**

^a^Functional genes correlated significantly (*P* < 0.01) with aboveground biomass are presented in bold.

^b^The carbon degradation genes include *amyA* (alpha amylase), *ara* (arabinofuranosidase), *xylA* (xylose isomerase), *vanA* (vanillate monooxygenase), *aceB* (malate synthase), *chi* (endochitinase), *lip* (ligninase) and acetylglucosaminidase; and the N cyling genes include*ureC* (urease), *nirK* (nitrite reductase), *nosZ* (nitrous oxide reductase), *napA* (nitrate reductase), *nrfA* (c-type cytochrome nitrite reductase) and *nifH* (nitrogenase reductase).

**Table 4:** Major topological properties of functional molecular ecological networks (fMENs) of soil microbial communities under non-planting and planting treatments.

Community	No. of original genes^a^	Similarity threshold (S_t_)	Network size (n)^b^	Total links	Average connectivity (avgK)	Modularity (no. of modules)
Non-planting (whole)	612	0.95	348	671	3.86	0.80 (53)
Planting (whole)	2829	0.96	1834	7585	8.27	0.89 (169)
Non-planting (C)	100	0.88	58	56	1.93	0.80 (16)
Planting (C)	470	0.89	354	911	5.15	0.83 (25)
Non-planting (N)	78	0.86	45	82	3.64	0.39(8)
Planting (N)	318	0.86	272	634	4.66	0.79(28)

^a^The total number of genes used for constructing a functional molecular ecological network.

^b^The total number of nodes in a functional molecular ecological network.
