# Supplementary material for: Planting increases the abundance and structure complexity of soil core functional genes relevant to carbon and nitrogen cycling
Source: Sci Rep. 2015 Sep 23;5:14345. doi: 10.1038/srep14345 (PMC4585818; doi:10.1038/srep14345)
Supplement: Supplementary Information [file srep14345-s1.pdf]

Supplementary information

Tables S1-S5, Figures S1-S5

Planting increases the abundance and structure complexity of soil core functional genes relevant to carbon and nitrogen cycling

Feng Wang[1, 2 †], Yuting Liang[1†], Yuji Jiang[1], Yunfeng Yang[3], Kai Xue[4], Jinbo Xiong[4], Jizhong Zhou[3, 4, 5], Bo Sun [1*]

[1] *State Key Laboratory of Soil and Sustainable Agriculture, Institute of Soil Science, Chinese Academy of Sciences, No. 71 East Beijing Road, Nanjing 210008, China*

[2] *Ningbo Academy of Agricultural Sciences, No. 19 Dehou Street, Ningbo 315040, China*

[3] *State Key Joint Laboratory of Environment Simulation and Pollution Control, School of Environment, Tsinghua University, Beijing 100084, China*

[4] *Institute for Environmental Genomics and Department of Botany and Microbiology, University of Oklahoma, Norman, OK 73019, USA*

[5] *Earth Sciences Division, Lawrence Berkeley National Laboratory, Berkeley, CA 94720, USA*

[†]Both contributed equally.

[*]Corresponding author: Tel.: +86 25 86881282; fax: +86 25 86881000.

E-mail address: bsun@issas.ac.cn (B. Sun).

Table S1

Dissimilarity tests (adonis) of effects of planting on functional gene community structure and environmental variables among three sites

	Functional gene community			*Environment variables* [*]		
	N	M	S	N	M	S
R^2	0.738	0.798	0.913	0.928	0.975	0.999
P	0.048	0.040	<0.001	0.059	0.035	0.012

[*] Environment variables included soil (pH, SOM, TN, TP, TK, AN, AP, AK, NH_4-N, NO_3-N, mean soil temperature, mean soil moisture) and plant variables (aboveground biomass, SPAD). The distance was calculated by Bray-Curtis method in permutational multivariate analysis of variance.

Table S2

Core functional genes involving in carbon degradataion attributed to planting effects

Genbank. ID	Gene	Sub-category	Organism
118462569	CDH	Cellulose	Mycobacterium avium 104
110825410	CDH	Cellulose	Rhodococcus sp. RHA1
304359	bgl	Cellulose	Cellulomonas fimi
151362421	bgl	Cellulose	Kineococcus radiotolerans SRS30216
116671523	bgl	Cellulose	Arthrobacter sp. FB24
73698632	bgl	Cellulose	Alicyclobacillus acidocaldarius
58337637	bgl	Cellulose	Lactobacillus acidophilus NCFM
142230	bgl	Cellulose	Agrobacterium sp.
99078285	bgl	Cellulose	Silicibacter sp. TM1040
153892922	bgl	Cellulose	Opitutaceae bacterium TAV2
15023816	bcsG	Cellulose	Clostridium acetobutylicum ATCC 824
31747164	bcsG	Cellulose	Hypocrea jecorina
7804883	exoglucanase	Cellulose	Leptosphaeria maculans
7107367	exoglucanase	Cellulose	Trichoderma harzianum
29160303	exoglucanase	Cellulose	Acremonium thermophilum
157362170	exoglucanase	Cellulose	Polyporus arcularius
25990955	exoglucanase	Cellulose	Piromyces sp. E2
108764717	acetylglucosaminidase	Chitin	Rubrobacter xylanophilus DSM 9941
85831632	acetylglucosaminidase	Chitin	Leeuwenhoekiella blandensis MED217
83815976	acetylglucosaminidase	Chitin	Salinibacter ruber DSM 13855
116253959	acetylglucosaminidase	Chitin	Rhizobium leguminosarum bv. viciae 3841
119774080	acetylglucosaminidase	Chitin	Shewanella amazonensis SB2B
118071251	acetylglucosaminidase	Chitin	Shewanella woodyi ATCC 51908
46912654	acetylglucosaminidase	Chitin	Photobacterium profundum SS9
133778506	chi	Chitin	uncultured bacterium
133778504	chi	Chitin	uncultured bacterium
113945922	chi	Chitin	Salinispora tropica CNB-440
145595773	chi	Chitin	Salinispora tropica CNB-440
145595484	chi	Chitin	Salinispora tropica CNB-440
85813892	chi	Chitin	Streptomyces rimosus subsp. paromomycinus
149279875	chi	Chitin	Pedobacter sp. BAL39

142688	chi	Chitin	Bacillus circulans
46881385	chi	Chitin	Listeria monocytogenes str. 4b F2365
111617776	chi	Chitin	Acidovorax avenae subsp. citrulli AAC00-1
120590559	chi	Chitin	Acidovorax avenae subsp. citrulli AAC00-1
115375191	chi	Chitin	Stigmatella aurantiaca DW4/3-1
108460873	chi	Chitin	Myxococcus xanthus DK 1622
108760660	chi	Chitin	Myxococcus xanthus DK 1622
88778030	chi	Chitin	Reinekea sp. MED297
142852981	chi	Chitin	Aeromonas salmonicida subsp. salmonicida A449
18143418	chi	Chitin	Alteromonas sp. O-7
83631795	chi	Chitin	Hahella chejuensis KCTC 2396
70729461	chi	Chitin	Pseudomonas fluorescens Pf-5
66048001	chi	Chitin	Pseudomonas syringae pv. syringae B728a
151939785	chi	Chitin	Vibrio sp. Ex25
90580479	chi	Chitin	Vibrio angustum S14
156529061	chi	Chitin	Vibrio harveyi ATCC BAA-1116
145229305	chi	Chitin	Aspergillus niger
145257729	chi	Chitin	Aspergillus niger
119189427	chi	Chitin	Coccidioides immitis RS
113129062	chi	Chitin	Hypocrea virens
21223376	exochitinase	Chitin	Streptomyces coelicolor A3(2)
116623106	ara	Hemicellulose	Solibacter usitatus Ellin6076
116621213	ara	Hemicellulose	Solibacter usitatus Ellin6076
69284222	ara	Hemicellulose	Kineococcus radiotolerans SRS30216
116608903	ara	Hemicellulose	Arthrobacter sp. FB24
23326899	ara	Hemicellulose	Bifidobacterium longum NCC2705
149936337	ara	Hemicellulose	Parabacteroides distasonis ATCC 8503
85831189	ara	Hemicellulose	Leeuwenhoekiella blandensis MED217
106889690	ara	Hemicellulose	Roseiflexus sp. RS-1
156869730	ara	Hemicellulose	Clostridium leptum DSM 753
150385878	ara	Hemicellulose	Victivallis vadensis ATCC BAA-548
46205560	ara	Hemicellulose	Magnetospirillum magnetotacticum MS-1
156934116	ara	Hemicellulose	Enterobacter sakazakii ATCC BAA-894
21242039	ara	Hemicellulose	Xanthomonas axonopodis pv. citri str. 306
1244586	ara_fungi	Hemicellulose	Aspergillus niger

120577985	ara_fungi	Hemicellulose	Penicillium capsulatum
154304204	ara_fungi	Hemicellulose	Botryotinia fuckeliana B05.10
154297971	ara_fungi	Hemicellulose	Botryotinia fuckeliana B05.10
60416980	ara_fungi	Hemicellulose	Fusarium oxysporum f. sp. dianthi
116208630	ara_fungi	Hemicellulose	Chaetomium globosum CBS 148.51
106885867	mannanase	Hemicellulose	Clostridium phytofermentans ISDg
88814399	xylA	Hemicellulose	marine actinobacterium PHSC20C1
50950653	xylA	Hemicellulose	Leifsonia xyli subsp. xyli str. CTCB07
141994	xylA	Hemicellulose	Actinoplanes sp. ATCC 31351
145596104	xylA	Hemicellulose	Salinispora tropica CNB-440
71368759	xylA	Hemicellulose	Nocardioides sp. JS614
88710156	xylA	Hemicellulose	Flavobacteriales bacterium HTCC2170
156742580	xylA	Hemicellulose	Roseiflexus castenholzii DSM 13941
94554210	xylA	Hemicellulose	Deinococcus geothermalis DSM 11300
114706526	xylA	Hemicellulose	Fulvimarina pelagi HTCC2506
37526188	xylA	Hemicellulose	Photorhabdus luminescens subsp. laumondii TTO1
23304849	xyn	Hemicellulose	Clostridium stercorarium
106887012	xyn	Hemicellulose	Clostridium phytofermentans ISDg
113935746	xyn	Hemicellulose	Caulobacter sp. K31
13422062	xyn	Hemicellulose	Caulobacter crescentus CB15
86282167	xyn	Hemicellulose	Rhizobium etli CFN 42
113876349	xyn	Hemicellulose	Sinorhizobium medicae WSM419
103488518	xyn	Hemicellulose	Sphingopyxis alaskensis RB2256
89950900	xyn	Hemicellulose	Saccharophagus degradans 2-40
70986823	xyn	Hemicellulose	Aspergillus fumigatus Af293
154299780	glx	Lignin	Botryotinia fuckeliana B05.10
89113933	glx	Lignin	Phanerochaete chrysosporium
58268820	glx	Lignin	Cryptococcus neoformans var. neoformans JEC21
46096440	glx	Lignin	Ustilago maydis 521
598355	lip	Lignin	Phanerochaete chrysosporium
5616325	lip	Lignin	Ceriporiopsis subvermispora
40714547	lip	Lignin	Trametes versicolor
126105557	phenol_oxidase	Lignin	uncultured fungus
126105533	phenol_oxidase	Lignin	uncultured fungus
126035159	phenol_oxidase	Lignin	uncultured fungus

126105519	phenol_oxidase	Lignin	uncultured fungus
145236308	phenol_oxidase	Lignin	Aspergillus niger
120431228	phenol_oxidase	Lignin	Sclerotinia minor
152013640	phenol_oxidase	Lignin	Fusarium oxysporum
37703775	phenol_oxidase	Lignin	Coprinopsis cinerea
115371535	phenol_oxidase	Lignin	Coprinopsis cinerea okayama7#130
56785440	phenol_oxidase	Lignin	Trametes sp. AH28-2
2264396	phenol_oxidase	Lignin	basidiomycete CECT 20197
121495873	phenol_oxidase	Lignin	Ganoderma lucidum
124495022	phenol_oxidase	Lignin	Polyporus brumalis
114228535	phenol_oxidase	Lignin	Cryptococcus neoformans var. grubii
88815516	aceA	Others	marine actinobacterium PHSC20C1
119692341	aceA	Others	Mycobacterium sp. KMS
222527412	aceA	Others	Chloroflexus sp. Y-400-fl
6458545	aceA	Others	Deinococcus radiodurans R1
10175298	aceA	Others	Bacillus halodurans C-125
74422036	aceA	Others	Nitrobacter winogradskyi Nb-255
150395535	aceA	Others	Sinorhizobium medicae WSM419
124267078	aceA	Others	Methylibium petroleiphilum PM1
197123496	aceA	Others	Anaeromyxobacter sp. K
121589589	aceA	Others	Halorhodospira halophila SL1
121998184	aceA	Others	Halorhodospira halophila SL1
88789809	aceA	Others	Nitrococcus mobilis Nb-231
88812887	aceA	Others	Nitrococcus mobilis Nb-231
195941139	aceA	Others	Escherichia coli O157:H7 str. EC4024
50122910	aceA	Others	Pectobacterium atrosepticum SCRI1043
161505331	aceA	Others	Salmonella enterica subsp. arizonae serovar 62:z4,z23:--
90440222	aceA	Others	Vibrio angustum S14
194347636	aceA	Others	Stenotrophomonas maltophilia R551-3
121703954	aceA	Others	Aspergillus clavatus NRRL 1
70993266	aceA	Others	Aspergillus fumigatus Af293
212534768	aceA	Others	Penicillium marneffei ATCC 18224
49651987	aceA	Others	Yarrowia lipolytica CLIB122
1314081	aceA	Others	Saccharomyces cerevisiae
145702065	aceB	Others	Metallosphaera sedula DSM 5348

228009109	aceB	Others	Sulfolobus islandicus Y.G.57.14
18312527	aceB	Others	Pyrobaculum aerophilum str. IM2
126249557	aceB	Others	Pyrobaculum calidifontis JCM 11548
226870051	aceB	Others	Actinosynnema mirum DSM 43827
25028787	aceB	Others	Corynebacterium efficiens YS-314
227079376	aceB	Others	Corynebacterium lipophiloflavum DSM 44291
119949946	aceB	Others	Arthrobacter aurescens TC1
54312035	aceB	Others	Mycobacterium sp. P101
145216439	aceB	Others	Mycobacterium gilvum PYR-GCK
197703172	aceB	Others	Streptomyces clavuligerus ATCC 27064
178463953	aceB	Others	Streptomyces griseus subsp. griseus NBRC 13350
108766127	aceB	Others	Rubrobacter xylanophilus DSM 9941
156744289	aceB	Others	Roseiflexus castenholzii DSM 13941
159897545	aceB	Others	Herpetosiphon aurantiacus ATCC 23779
15806175	aceB	Others	Deinococcus radiodurans R1
46196060	aceB	Others	Thermus thermophilus HB27
221089232	aceB	Others	Geobacillus sp. Y412MC61
220706797	aceB	Others	Geobacillus sp. Y412MC61
196248380	aceB	Others	Geobacillus sp. G11MC16
51855416	aceB	Others	Symbiobacterium thermophilum IAM 14863
16126009	aceB	Others	Caulobacter crescentus CB15
84704542	aceB	Others	Parvularcula bermudensis HTCC2503
84689378	aceB	Others	Parvularcula bermudensis HTCC2503
182678865	aceB	Others	Beijerinckia indica subsp. indica ATCC 9039
182634735	aceB	Others	Beijerinckia indica subsp. indica ATCC 9039
209874700	aceB	Others	Oligotropha carboxidovorans OM5
14024664	aceB	Others	Mesorhizobium loti MAFF303099
190694973	aceB	Others	Rhizobium etli CIAT 652
86355720	aceB	Others	Rhizobium etli CFN 42
150398462	aceB	Others	Sinorhizobium medicae WSM419
84516932	aceB	Others	Loktanella vestfoldensis SKA53
84391670	aceB	Others	Oceanicola batsensis HTCC2597
89067960	aceB	Others	Oceanicola granulosus HTCC2516
77462527	aceB	Others	Rhodobacter sphaeroides 2.4.1
126740332	aceB	Others	Roseobacter sp. SK209-2-6

225133279	aceB	Others	Roseobacter sp. GAI101
109456619	aceB	Others	Roseobacter denitrificans OCh 114
85703610	aceB	Others	Roseovarius sp. 217
114540602	aceB	Others	Roseovarius sp. HTCC2601
56679414	aceB	Others	Ruegeria pomeroyi DSS-3
170142908	aceB	Others	Burkholderia graminis C4D1M
120587575	aceB	Others	Acidovorax avenae subsp. citrulli AAC00-1
91699574	aceB	Others	Polaromonas sp. JS666
133738798	aceB	Others	Herminiimonas arsenicoxydans
78218626	aceB	Others	Desulfovibrio desulfuricans G20
218758227	aceB	Others	Desulfovibrio vulgaris str. 'Miyazaki F'
153005821	aceB	Others	Anaeromyxobacter sp. Fw109-5
225085216	aceB	Others	gamma proteobacterium NOR51-B
88799326	aceB	Others	Reinekea sp. MED297
90413673	aceB	Others	Photobacterium profundum 3TCK
151938860	aceB	Others	Vibrio sp. Ex25
225142176	aceB	Others	Stenotrophomonas sp. SKA14
212527146	aceB	Others	Penicillium marneffei ATCC 18224
154277552	aceB	Others	Ajellomyces capsulatus NAm1
114228552	aceB	Others	Paracoccidioides brasiliensis
145609990	aceB	Others	Magnaporthe grisea 70-15
16308743	aceB	Others	Laccaria bicolor
58270612	aceB	Others	Cryptococcus neoformans var. neoformans JEC21
111609818	AssA	Others	sulfate-reducing bacterium AK-01
iegAssA27	AssA	Others	Unknown
iegAssA7	AssA	Others	Unknown
118470516	limEH	Aromatics	Mycobacterium smegmatis str. MC2 155
90195860	limEH	Aromatics	Mycobacterium vanbaalenii PYR-1
54026163	limEH	Aromatics	Nocardia farcinica IFM 10152
113731798	limEH	Aromatics	Caulobacter sp. K31
114050057	vanA	Aromatics	Streptomyces ambofaciens
75701251	vanA	Aromatics	Anabaena variabilis ATCC 29413
113733254	vanA	Aromatics	Caulobacter sp. K31
27350645	vanA	Aromatics	Bradyrhizobium japonicum USDA 110
109455731	vanA	Aromatics	Roseobacter denitrificans OCh 114

148501358	vanA	Aromatics	Sphingomonas wittichii RW1
148502944	vanA	Aromatics	Sphingomonas wittichii RW1
110831199	vanA	Aromatics	Verminephrobacter eiseniae EF01-2
149814105	vanA	Aromatics	Plesiocystis pacifica SIR-1
119459477	vanA	Aromatics	marine gamma proteobacterium HTCC2080
91795559	vanA	Aromatics	Chromohalobacter salexigens DSM 3043
119816076	vanA	Aromatics	Pseudomonas putida W619
120592900	vdh	Aromatics	Polaromonas naphthalenivorans CJ2
10184710	vdh	Aromatics	Pseudomonas sp. HR199
66847744	vdh	Aromatics	Aspergillus fumigatus Af293
59044731	pectinase	Pectin	uncultured bacterium
166516	pectinase	Pectin	Aspergillus niger
2251108	amyA	Starch	Pyrococcus sp.
48306	amyA	Starch	unidentified bacterium
68234636	amyA	Starch	Frankia sp. EAN1pec
86566918	amyA	Starch	Frankia sp. CcI3
92441508	amyA	Starch	Mycobacterium sp. KMS
72161237	amyA	Starch	Thermobifida fusca YX
72162290	amyA	Starch	Thermobifida fusca YX
50842594	amyA	Starch	Propionibacterium acnes KPA171202
119026577	amyA	Starch	Bifidobacterium adolescentis ATCC 15703
156861181	amyA	Starch	Bacteroides uniformis ATCC 8492
7190353	amyA	Starch	Chlamydia muridarum Nigg
76164999	amyA	Starch	Chloroflexus aurantiacus J-10-fl
76260074	amyA	Starch	Chloroflexus aurantiacus J-10-fl
87303008	amyA	Starch	Synechococcus sp. WH 5701
75907651	amyA	Starch	Anabaena variabilis ATCC 29413
119465693	amyA	Starch	Nodularia spumigena CCY9414
119493471	amyA	Starch	Lyngbya sp. PCC 8106
33238381	amyA	Starch	Prochlorococcus marinus subsp. marinus str. CCMP1375
6457937	amyA	Starch	Deinococcus radiodurans R1
89098045	amyA	Starch	Bacillus sp. NRRL B-14911
46487401	amyA	Starch	Bacillus halodurans
52784460	amyA	Starch	Bacillus licheniformis ATCC 14580
29343376	amyA	Starch	Enterococcus faecalis V583

28270020	amyA	Starch	Lactobacillus plantarum WCFS1
90821679	amyA	Starch	Lactobacillus salivarius subsp. salivarius UCC118
82744605	amyA	Starch	Clostridium beijerinckii NCIMB 8052
89210693	amyA	Starch	Halothermothrix orenii H 168
27376012	amyA	Starch	Bradyrhizobium japonicum USDA 110
91977680	amyA	Starch	Rhodopseudomonas palustris BisB5
86572402	amyA	Starch	Rhodopseudomonas palustris HaA2
157408057	amyA	Starch	Methylobacterium populi BJ001
85373461	amyA	Starch	Erythrobacter litoralis HTCC2594
88936984	amyA	Starch	Geobacter uraniumreducens Rf4
85777065	amyA	Starch	Anaeromyxobacter dehalogenans 2CP-C
88798833	amyA	Starch	Reinekea sp. MED297
119768086	amyA	Starch	Shewanella amazonensis SB2B
49613597	amyA	Starch	Erwinia carotovora subsp. atroseptica SCRI1043
50121937	amyA	Starch	Erwinia carotovora subsp. atroseptica SCRI1043
77963046	amyA	Starch	Yersinia mollaretii ATCC 43969
1177849	amyA	Starch	Pseudomonas sp. KFCC10818
156528074	amyA	Starch	Vibrio harveyi ATCC BAA-1116
85104727	amyA	Starch	Neurospora crassa OR74A
10175887	amyX	Starch	Bacillus halodurans C-125
2788	glucoamylase	Starch	Amorphotheca resinae
1905759	isopullulanase	Starch	Aspergillus niger
29605797	pulA	Starch	Streptomyces avermitilis MA-4680
153808537	pulA	Starch	Bacteroides caccae ATCC 43185
125497235	pulA	Starch	Streptococcus sanguinis SK36
15966601	pulA	Starch	Sinorhizobium meliloti 1021
83312163	pulA	Starch	Magnetospirillum magneticum AMB-1
67535763	pulA	Starch	Burkholderia vietnamiensis G4
116749778	pulA	Starch	Syntrophobacter fumaroxidans MPOB
119468775	pulA	Starch	Alteromonadales bacterium TW-7
4836215	pulA	Starch	Fervidobacterium pennivorans

Table S3

Core functional genes involving in nitrogen cycling attributed to planting effects

Genbank. ID	Gene	Sub-category	Organism
124007002	gdh	Ammonification	Microscilla marina ATCC 23134
146192311	gdh	Ammonification	Bradyrhizobium sp. ORS278
34494872	ureC	Ammonification	Haloarcula vallismortis
76557515	ureC	Ammonification	Natronomonas pharaonis DSM 2160
158317443	ureC	Ammonification	Frankia sp. EAN1pec
152964803	ureC	Ammonification	Kineococcus radiotolerans SRS30216
67985209	ureC	Ammonification	Kineococcus radiotolerans SRS30216
118618420	ureC	Ammonification	Mycobacterium ulcerans Agy99
113941017	ureC	Ammonification	Herpetosiphon aurantiacus ATCC 23779
158338215	ureC	Ammonification	Acaryochloris marina MBIC11017
42782712	ureC	Ammonification	Bacillus cereus ATCC 10987
21435824	ureC	Ammonification	Streptococcus alactolyticus
125714545	ureC	Ammonification	Clostridium thermocellum ATCC 27405
39650578	ureC	Ammonification	Rhodopseudomonas palustris CGA009
113725650	ureC	Ammonification	Sinorhizobium medicae WSM419
150029098	ureC	Ammonification	Sinorhizobium medicae WSM419
119383953	ureC	Ammonification	Paracoccus denitrificans PD1222
85669952	ureC	Ammonification	Roseovarius sp. 217
126708478	ureC	Ammonification	Sagittula stellata E-37
145047781	ureC	Ammonification	Polynucleobacter sp. QLW-P1DMWA-1
151575999	ureC	Ammonification	Ralstonia pickettii 12D
84693457	ureC	Ammonification	Polaromonas naphthalenivorans CJ2
37731823	ureC	Ammonification	Nitrosomonas cryotolerans
77166331	ureC	Ammonification	Nitrosococcus oceani ATCC 19707
77380811	ureC	Ammonification	Pseudomonas fluorescens PfO-1
121702879	ureC	Ammonification	Aspergillus clavatus NRRL 1
ieghzo270	hzo	Anammox	Unknown
71383860	nasA	Assimilatory N reduction	uncultured bacterium
155964459	nasA	Assimilatory N reduction	uncultured marine bacterium
116804538	nasA	Assimilatory N reduction	uncultured prokaryote
116804542	nasA	Assimilatory N reduction	uncultured prokaryote

227069639	nirA	Assimilatory N reduction	uncultured archaeon
227069699	nirA	Assimilatory N reduction	uncultured archaeon
227069643	nirA	Assimilatory N reduction	uncultured archaeon
172036138	nirA	Assimilatory N reduction	Cyanothece sp. ATCC 51142
171697592	nirA	Assimilatory N reduction	Cyanothece sp. ATCC 51142
166363013	nirA	Assimilatory N reduction	Microcystis aeruginosa NIES-843
86556078	nirA	Assimilatory N reduction	Synechococcus sp. JA-2-3B'a(2-13)
148255520	nirA	Assimilatory N reduction	Bradyrhizobium sp. BTAi1
182413555	nirA	Assimilatory N reduction	Opitutus terrae PB90-1
134101441	NirB	Assimilatory N reduction	Saccharopolyspora erythraea NRRL 2338
29832204	NirB	Assimilatory N reduction	Streptomyces avermitilis MA-4680
32471653	NirB	Assimilatory N reduction	Rhodopirellula baltica SH 1
209874150	NirB	Assimilatory N reduction	Oligotropha carboxidovorans OM5
213925440	NirB	Assimilatory N reduction	Pseudomonas syringae pv. tomato T1
94471203	narG	Denitrification	uncultured bacterium
38427020	narG	Denitrification	uncultured bacterium
158138839	narG	Denitrification	uncultured bacterium
157285559	narG	Denitrification	uncultured bacterium
124488337	narG	Denitrification	uncultured bacterium
26278828	narG	Denitrification	uncultured bacterium
94471271	narG	Denitrification	uncultured bacterium
45386161	narG	Denitrification	unidentified bacterium
62003549	narG	Denitrification	uncultured bacterium
26278922	narG	Denitrification	uncultured bacterium
32308029	narG	Denitrification	uncultured bacterium
94471277	narG	Denitrification	uncultured bacterium
45386181	narG	Denitrification	unidentified bacterium
94471349	narG	Denitrification	uncultured bacterium
119391415	narG	Denitrification	uncultured bacterium
62003537	narG	Denitrification	uncultured bacterium
121495608	narG	Denitrification	uncultured bacterium
94471237	narG	Denitrification	uncultured bacterium
29652532	narG	Denitrification	uncultured bacterium
32307929	narG	Denitrification	uncultured bacterium
45386179	narG	Denitrification	unidentified bacterium

29652552	narG	Denitrification	uncultured bacterium
76056963	narG	Denitrification	uncultured bacterium
66801911	narG	Denitrification	unidentified bacterium
94471133	narG	Denitrification	uncultured bacterium
26278800	narG	Denitrification	uncultured bacterium
26278878	narG	Denitrification	uncultured bacterium
45386203	narG	Denitrification	unidentified bacterium
158138865	narG	Denitrification	uncultured bacterium
78093554	narG	Denitrification	uncultured bacterium
119391597	narG	Denitrification	uncultured bacterium
28378213	narG	Denitrification	Lactobacillus plantarum WCFS1
34332835	narG	Denitrification	Chromobacterium violaceum ATCC 12472
56798123	narG	Denitrification	Pseudomonas sp. MT-1
112462695	nirK	Denitrification	uncultured bacterium
46409941	nirK	Denitrification	uncultured bacterium
112462135	nirK	Denitrification	uncultured bacterium
125971537	nirK	Denitrification	uncultured denitrifying bacterium
83316998	nirK	Denitrification	uncultured bacterium
87281050	nirK	Denitrification	uncultured bacterium
112463925	nirK	Denitrification	uncultured bacterium
73762870	nirK	Denitrification	uncultured bacterium
112463277	nirK	Denitrification	uncultured bacterium
83316708	nirK	Denitrification	uncultured bacterium
112462455	nirK	Denitrification	uncultured bacterium
73762890	nirK	Denitrification	uncultured bacterium
87281165	nirK	Denitrification	uncultured bacterium
112463637	nirK	Denitrification	uncultured bacterium
109632807	nirK	Denitrification	uncultured bacterium
17742853	nirK	Denitrification	Agrobacterium tumefaciens str. C58
28542629	nirS	Denitrification	uncultured bacterium
76577396	nirS	Denitrification	uncultured bacterium
74038408	nirS	Denitrification	uncultured bacterium
57335474	nirS	Denitrification	uncultured bacterium
51534865	nirS	Denitrification	uncultured bacterium
87281376	nirS	Denitrification	uncultured bacterium

68510354	nirS	Denitrification	uncultured bacterium
74038370	nirS	Denitrification	uncultured bacterium
116013320	nirS	Denitrification	uncultured bacterium
87281183	nirS	Denitrification	uncultured bacterium
28542625	nirS	Denitrification	uncultured bacterium
46850218	nirS	Denitrification	uncultured bacterium
83318864	nirS	Denitrification	uncultured bacterium
87281302	nirS	Denitrification	uncultured bacterium
28542639	nirS	Denitrification	uncultured bacterium
81251653	nirS	Denitrification	uncultured organism
81251657	nirS	Denitrification	uncultured organism
29466008	norB	Denitrification	uncultured bacterium
29466064	norB	Denitrification	uncultured bacterium
29466090	norB	Denitrification	uncultured bacterium
29466006	norB	Denitrification	uncultured bacterium
29466024	norB	Denitrification	uncultured bacterium
29466086	norB	Denitrification	uncultured bacterium
4454060	norB	Denitrification	Bradyrhizobium japonicum
34391456	norB	Denitrification	Nitrosomonas marina
119671792	norB	Denitrification	Azoarcus sp. BH72
11344614	norB	Denitrification	Pseudomonas fluorescens
29125948	nosZ	Denitrification	uncultured soil bacterium
4633560	nosZ	Denitrification	uncultured bacterium 696W
4633558	nosZ	Denitrification	uncultured bacterium ProE
29125958	nosZ	Denitrification	uncultured soil bacterium
12744271	nosZ	Denitrification	uncultured temperate forest soil bacterium CZ1441
76058152	nosZ	Denitrification	uncultured bacterium
156446963	nosZ	Denitrification	uncultured bacterium
4633565	nosZ	Denitrification	uncultured bacterium ProI
156446925	nosZ	Denitrification	uncultured bacterium
4633583	nosZ	Denitrification	uncultured bacterium 696G
83972352	nosZ	Denitrification	uncultured alpha proteobacterium
69938258	nosZ	Denitrification	Paracoccus denitrificans PD1222
74056902	nosZ	Denitrification	Thiobacillus denitrificans ATCC 25259
119863768	nosZ	Denitrification	Psychromonas ingrahamii 37

124488109	napA	Dissimilatory N reduction	uncultured bacterium
148474840	napA	Dissimilatory N reduction	uncultured bacterium
124488145	napA	Dissimilatory N reduction	uncultured bacterium
158138747	napA	Dissimilatory N reduction	uncultured bacterium
146195550	napA	Dissimilatory N reduction	Bradyrhizobium sp. ORS278
146406446	napA	Dissimilatory N reduction	Bradyrhizobium sp. BTAi1
38637875	napA	Dissimilatory N reduction	Cupriavidus necator
156255109	napA	Dissimilatory N reduction	Pseudomonas sp. MT-1
149196318	nrfA	Dissimilatory N reduction	Lentisphaera araneosa HTCC2155
94265724	nrfA	Dissimilatory N reduction	delta proteobacterium MLMS-1
95133480	nrfA	Dissimilatory N reduction	Desulfuromonas acetoxidans DSM 684
144945974	nrfA	Dissimilatory N reduction	Geobacter bemidjiensis Bem
77920456	nrfA	Dissimilatory N reduction	Pelobacter carbinolicus DSM 2380
86152755	nrfA	Dissimilatory N reduction	Campylobacter jejuni subsp. jejuni HB93-13
78365334	nrfA	Dissimilatory N reduction	Shewanella sp. PV-4
149191083	nrfA	Dissimilatory N reduction	Vibrio shilonii AK1
73697438	amoA	Nitrification	uncultured crenarchaeote
73697250	amoA	Nitrification	uncultured crenarchaeote
7595786	amoA	Nitrification	uncultured bacterium
71361353	amoA	Nitrification	uncultured bacterium
150014349	nifH	Nitrogen fixation	Methanococcus aeolicus Nankai-3
150400783	nifH	Nitrogen fixation	Methanococcus aeolicus Nankai-3
157401497	nifH	Nitrogen fixation	Methanococcus maripaludis C6
150399896	nifH	Nitrogen fixation	Methanococcus vannielii SB
154151622	nifH	Nitrogen fixation	Candidatus Methanoregula boonei 6A8
19917684	nifH	Nitrogen fixation	Methanosarcina acetivorans C2A
72394883	nifH	Nitrogen fixation	Methanosarcina barkeri str. fusaro
76667545	nifH	Nitrogen fixation	uncultured nitrogen-fixing bacterium
1255482	nifH	Nitrogen fixation	unidentified bacterium
29293450	nifH	Nitrogen fixation	uncultured bacterium
44829093	nifH	Nitrogen fixation	uncultured bacterium
139003462	nifH	Nitrogen fixation	uncultured nitrogen-fixing bacterium
3157524	nifH	Nitrogen fixation	unidentified nitrogen-fixing bacteria
60100873	nifH	Nitrogen fixation	uncultured bacterium
68135537	nifH	Nitrogen fixation	uncultured bacterium

99083279	nifH	Nitrogen fixation	uncultured bacterium
60100879	nifH	Nitrogen fixation	uncultured bacterium
3157614	nifH	Nitrogen fixation	unidentified nitrogen-fixing bacteria
110931976	nifH	Nitrogen fixation	uncultured marine bacterium
780709	nifH	Nitrogen fixation	unidentified marine eubacterium
148756010	nifH	Nitrogen fixation	uncultured bacterium
139004122	nifH	Nitrogen fixation	uncultured nitrogen-fixing bacterium
89512768	nifH	Nitrogen fixation	uncultured nitrogen-fixing bacterium
497884	nifH	Nitrogen fixation	uncultured nitrogen-fixing bacterium
62149130	nifH	Nitrogen fixation	uncultured bacterium
139003137	nifH	Nitrogen fixation	uncultured nitrogen-fixing bacterium
85069178	nifH	Nitrogen fixation	uncultured bacterium
99082987	nifH	Nitrogen fixation	uncultured bacterium
139003556	nifH	Nitrogen fixation	uncultured nitrogen-fixing bacterium
139003319	nifH	Nitrogen fixation	uncultured nitrogen-fixing bacterium
112280450	nifH	Nitrogen fixation	uncultured bacterium
76667400	nifH	Nitrogen fixation	uncultured nitrogen-fixing bacterium
156600794	nifH	Nitrogen fixation	uncultured bacterium
99083269	nifH	Nitrogen fixation	uncultured bacterium
73534169	nifH	Nitrogen fixation	uncultured bacterium
94470897	nifH	Nitrogen fixation	uncultured bacterium
60326762	nifH	Nitrogen fixation	uncultured nitrogen-fixing bacterium
89512616	nifH	Nitrogen fixation	uncultured nitrogen-fixing bacterium
56809495	nifH	Nitrogen fixation	uncultured bacterium
139003172	nifH	Nitrogen fixation	uncultured nitrogen-fixing bacterium
139004179	nifH	Nitrogen fixation	uncultured nitrogen-fixing bacterium
1255504	nifH	Nitrogen fixation	unidentified bacterium
76667453	nifH	Nitrogen fixation	uncultured nitrogen-fixing bacterium
148342406	nifH	Nitrogen fixation	uncultured bacterium
76667345	nifH	Nitrogen fixation	uncultured nitrogen-fixing bacterium
1255480	nifH	Nitrogen fixation	unidentified bacterium
10863143	nifH	Nitrogen fixation	marine stromatolite eubacterium HB(0697) A101A
139002666	nifH	Nitrogen fixation	uncultured nitrogen-fixing bacterium
99083075	nifH	Nitrogen fixation	uncultured bacterium
99083367	nifH	Nitrogen fixation	uncultured bacterium

99083095	nifH	Nitrogen fixation	uncultured bacterium
139003470	nifH	Nitrogen fixation	uncultured bacterium
37548710	nifH	Nitrogen fixation	uncultured bacterium
19070155	nifH	Nitrogen fixation	uncultured nitrogen-fixing bacterium
37925029	nifH	Nitrogen fixation	uncultured nitrogen-fixing bacterium
139003062	nifH	Nitrogen fixation	uncultured nitrogen-fixing bacterium
139003446	nifH	Nitrogen fixation	uncultured nitrogen-fixing bacterium
3157590	nifH	Nitrogen fixation	unidentified nitrogen-fixing bacteria
89512372	nifH	Nitrogen fixation	uncultured nitrogen-fixing bacterium
56809455	nifH	Nitrogen fixation	uncultured bacterium
29293252	nifH	Nitrogen fixation	uncultured bacterium
50919564	nifH	Nitrogen fixation	uncultured nitrogen-fixing bacterium
15186893	nifH	Nitrogen fixation	uncultured bacterium
89512930	nifH	Nitrogen fixation	uncultured nitrogen-fixing bacterium
151361599	nifH	Nitrogen fixation	Kineococcus radiotolerans SRS30216
147832086	nifH	Nitrogen fixation	Clavibacter michiganensis subsp. michiganensis NCPPB 382
78167184	nifH	Nitrogen fixation	Pelodictyon luteolum DSM 273
148567463	nifH	Nitrogen fixation	Roseiflexus sp. RS-1
16332205	nifH	Nitrogen fixation	Synechocystis sp. PCC 6803
138897063	nifH	Nitrogen fixation	Geobacillus thermodenitrificans NG80-2
2897667	nifH	Nitrogen fixation	Paenibacillus macerans
116812179	nifH	Nitrogen fixation	Clostridium sp. Sukashi-1
116812183	nifH	Nitrogen fixation	Clostridium sp. Kas107-2
33385703	nifH	Nitrogen fixation	Desulfotomaculum nigrificans
312237	nifH	Nitrogen fixation	Rhodobacter capsulatus
146278697	nifH	Nitrogen fixation	Rhodobacter sphaeroides ATCC 17025
85672433	nifH	Nitrogen fixation	Roseovarius sp. 217
158510468	nifH	Nitrogen fixation	Candidatus Desulfococcus oleovorans Hxd3
46562231	nifH	Nitrogen fixation	Desulfovibrio vulgaris subsp. vulgaris str. Hildenborough
118746565	nifH	Nitrogen fixation	Geobacter lovleyi SZ
116697525	nifH	Nitrogen fixation	Syntrophobacter fumaroxidans MPOB
34483462	nifH	Nitrogen fixation	Wolinella succinogenes
118072158	nifH	Nitrogen fixation	Shewanella woodyi ATCC 51908
12659200	nifH	Nitrogen fixation	Treponema sp. ZAS-9
12659196	nifH	Nitrogen fixation	Treponema primitia ZAS-2

Table S4. Core functional genes involving in carbon degradation and nitrogen cycling significantly (*P* < 0.01) correlated with aboveground biomass

Genbank. ID	Gene	Category	Organism
156529061	chi	Chitin degradation	Vibrio harveyi ATCC BAA-1116
145229305	chi	Chitin degradation	Aspergillus niger
145257729	chi	Chitin degradation	Aspergillus niger
113129062	chi	Chitin degradation	Hypocrea virens
113935746	xyn	Hemicellulose degradation	Caulobacter sp. K31
126105519	phox	Lignin degradation	uncultured fungus
156744289	aceB	Others degradation	Roseiflexus castenholzii DSM 13941
133738798	aceB	Others degradation	Herminiimonas arsenicoxydans
6457937	amyA	Starch degradation	Deinococcus radiodurans R1
67535763	pulA	Starch degradation	Burkholderia vietnamiensis G4
150029098	ureC	Ammonification	Sinorhizobium medicae WSM419
124488109	napA	Dissimilatory N reduction	uncultured bacterium
95133480	nrfA	Dissimilatory N reduction	Desulfuromonas acetoxidans DSM 684
3157614	nifH	Nitrogen fixation	unidentified nitrogen-fixing bacteria
148756010	nifH	Nitrogen fixation	uncultured bacterium
148342406	nifH	Nitrogen fixation	uncultured bacterium
19070155	nifH	Nitrogen fixation	uncultured nitrogen-fixing bacterium
2897667	nifH	Nitrogen fixation	Paenibacillus macerans

Table S5

Climate and soil conditions in three research sites

Site	Location	Climate type	MAT (°C) *	MAP (mm)	Soil type	Soil texture
North site: Hailun (N)	47°26' N, 126°38' E	cold temperate	3.0	530	Black soil (Phaeozem)	silty clay loam
Middle site: Fenqiu (M)	35°00' N, 114°24' E	warm temperate	13.9	605	Chao soil (Cambisol)	sandy loam
South site: Yingtan (S)	28°15N', 116°55' E	subtropical	17.6	1795	Red soil (Acrisol)	silty clay

*MAT (mean annual temperature) and MAP (mean precipitation data) were calculated from meteorological observation data from each experiment stations (1996-2006).

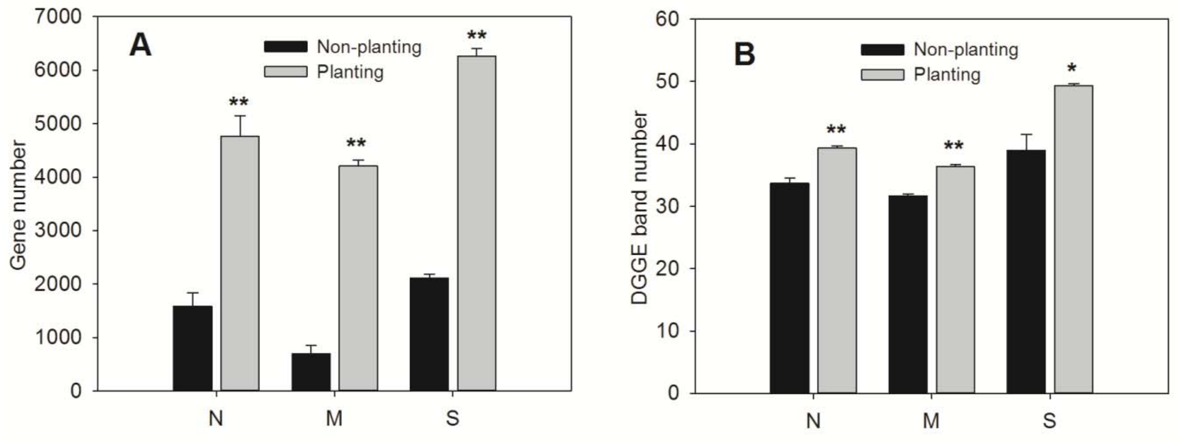

Figure S1. Total gene number detected by GeoChip 3.0 (A) and total DGGE band number (B) in three soilsunder non-planting and planting treatments.All data are presented as mean ± SE. The asterisk (*) and double asterisk (**) denote significant differences at $P< 0.05$ and $P <0.01$, respectively, which were assessed using two-tailed t test. N, north site in Hailun with cold temperate climate; M, middle site in Fengqiu with warm temperate climate; S, South site in Yingtan with subtropical climate.

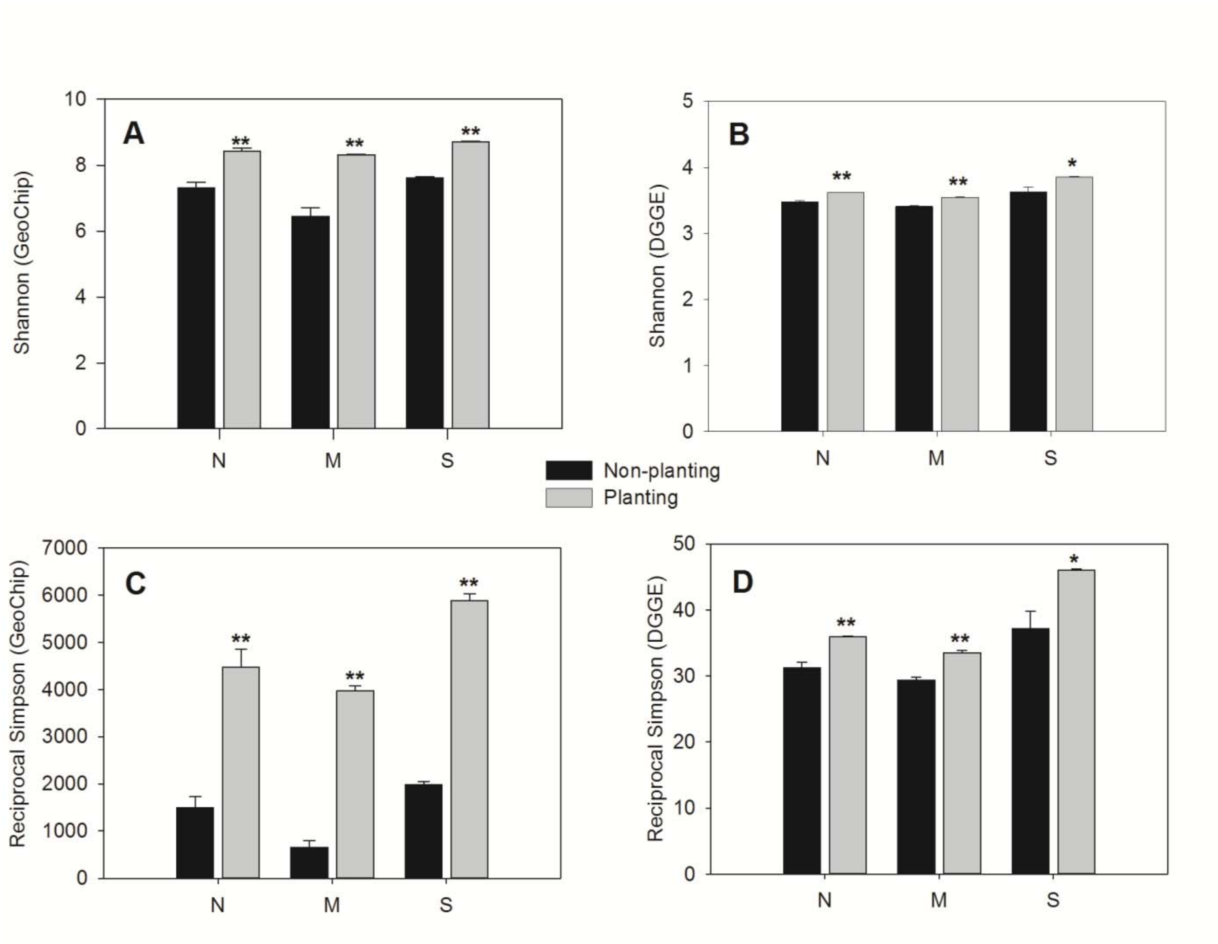

Figure S2. Shannon's and Simpson'sindexes of soil microbial community based on GeoChip and DGGE analysis in three soils under non-planting and planting treatments.All data are presented as mean ± SE. The asterisk (*) and double asterisk (**) denote significant differences at P< 0.05 and P <0.01, respectively, which was assessed using two-tailed t test. N, north site in Hailun with cold temperate climate; M, middle site in Fengqiu with warm temperate climate; S, South site in Yingtan with subtropical climate.

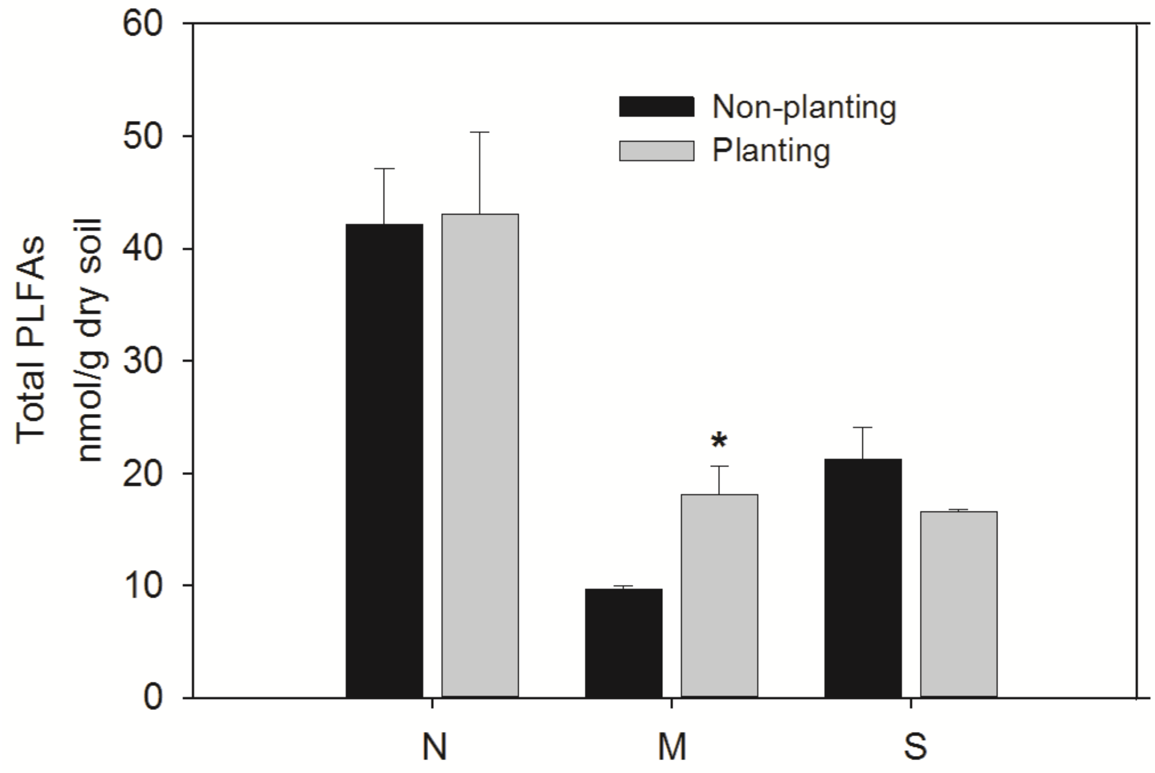

Figure S3. Total amounts of PLFAs in three soils under non-planting and planting treatments. All data are presented as mean±SE. The asterisk (*) denotes a significant differences at *P*< 0.05 which was assessed using two-tailed t test. N, north site in Hailun with cold temperate climate; M, middle site in Fengqiu with warm temperate climate; S, South site in Yingtan with subtropical climate.

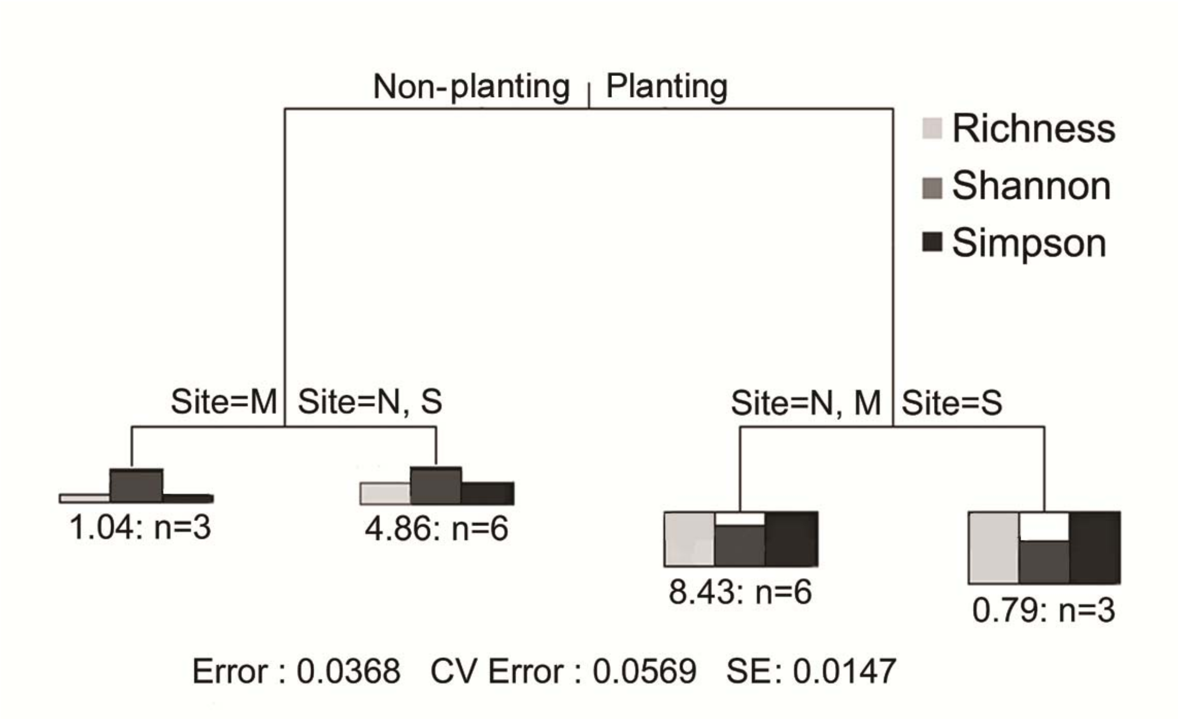

Figure S4. Multivariate regression tree (MRT) analysis for associations between α-diversities of soil functional genes and different treatments and locations. Bar plots show the multivariate means of diversity estimates at each branch, and the numbers of soil samples included in that splits are shown under bar plots. Error, relative error (it gives an over-optimistic estimate of how accurately a tree will predict for data); CV Error, cross-validated relative error (predictive accuracy is better estimated from the CV Error, which varies from zero for a perfect predictor to close to one for a poor predictor); SE, standard error. N, north site in Hailun with cold temperate climate; M, middle site in Fengqiu with warm temperate climate; S, South site in Yingtan with subtropical climate.

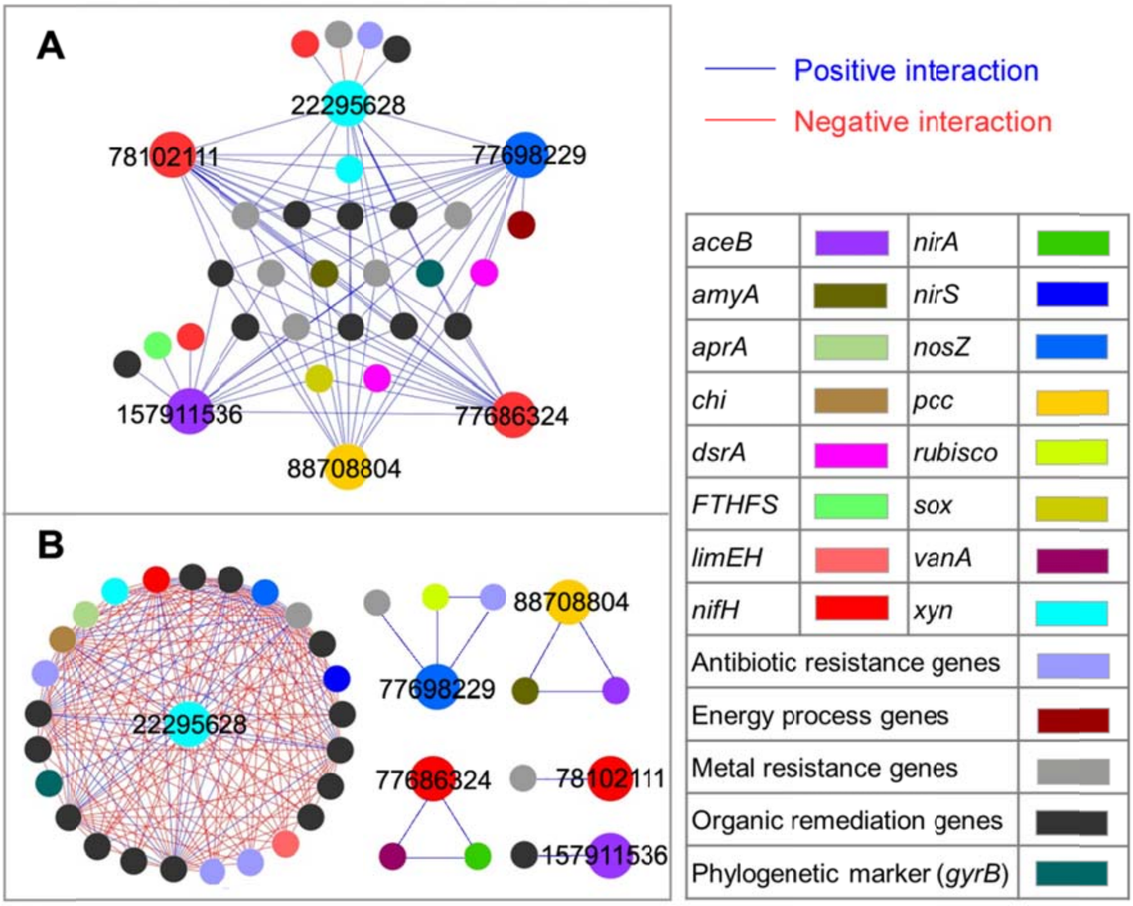

Figure S5. Network interactions of the top six functional genes with the highest connectivity under non-planting (A) and planting treatments (B). Each node signifies a functional gene/groups. Colors of the nodes indicate different functional genes/groups. A blue line indicates a positive interaction between two individual nodes, while a red line indicates a negative interaction. The listed genes in two networks under non-planting and planting treatments include *aceB* (malate synthase), *amyA* (alpha amylase), *chi* (endochitinase), *FTHFS* (tetrahydrofolate formylase), *limEH* (limonene epoxide hydrolase), *pcc* (propionyl-CoA carboxylase), *rubisco* (ribulose-l, 5-bisphosphate carboxylase/oxygenase), *vanA* (vanillate monooxygenase) and *xyn* (xylanase) for C cycling; *nifH* (nitrogenase reductase), *nirA* (nitrite reductase), *nirS* (nitrite reductase) and *nosZ*

(nitrous oxide reductase) for N cyxling; *aprA* (dissimilatory adenosine-5'-phospo-sulfate reductase), *dsrA* (Dissimilatory sulfite reductase) and *sox* (sulfite oxidase) for S cycling.
